# Smart microfluidic devices integrated in electrochemical point-of-care platforms for biomarker detection in biological fluids

**DOI:** 10.1007/s00216-025-06127-0

**Published:** 2025-10-14

**Authors:** Vincenzo Mazzaracchio, Fabiana Arduini

**Affiliations:** 1 Department of Chemical Science and Technologies, University of Rome “Tor Vergata”, Via Della Ricerca Scientifica 1, 00133 Rome, Italy; 2 SENSE4MED, Via Bitonto 139, 00133 Rome, Italy

**Keywords:** Microfluidic technologies, Electrochemical sensing, Point-of-care platforms, Biomarker detection, Wearable sensors

## Abstract

The increasing demand for decentralized, accessible, and rapid analytical tools is driving a transformation in healthcare toward point-of-care (POC) analytical technologies. The final aim is to reduce the cost of healthcare management originating from frequent patient hospitalizations and expensive and time-consuming laboratory-based analyses. This review explores the integration of microfluidic technologies with electrochemical sensing platforms, aiming to address the urgent need for POC analytical platforms. Owing to the miniaturization of fluid management systems and exploiting fluid automation, microfluidic devices enable low sample consumption, cost-effective analysis, and multiplexed detection, offering promising tools for real-time health monitoring. Among the other materials, the most commonly used substrates for microfluidics fabrication are paper, PDMS, and adhesive tape, which support custom-designed microchannel architectures, passive fluid motion, and wearable integration. Special attention is given to wearable sensors for sweat analysis, with various approaches employing capillary-driven flows and smart microfluidic designs to enable continuous and autonomous monitoring of biomarkers. Highlighting relevant works from the last 5 years, the review explores the role of integrated microfluidic electrochemical sensing devices in delivering advanced decentralized analytical platforms, with significant potential for clinical use in biomarker detection.

**Graphical abstract:**

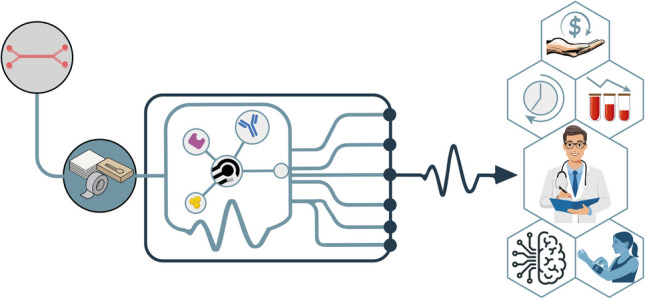

## Introduction

Healthcare management is entering a new era that underscores the importance of reducing hospitalization and extended inpatient stays [1, 2]. This shift is primarily driven by the high costs associated with hospital expenditures and frequent patient revisits [3], highlighting the urgent need for advanced analytical devices. These devices must be capable of performing decentralized, accurate, and user-friendly early disease detection/monitoring to ensure reliable patient outcomes. Prehospital and in-house diagnostic solutions, such as point-of-care (POC) tests, can meet these critical needs, as they are designed to align with the “ASSURED” criteria established by the World Health Organization. These criteria outline the essential characteristics of an ideal diagnostic test in resource-limited settings, where ASSURED stands for Affordable, Sensitive, Specific, User-friendly, Rapid, Equipment-free, and Deliverable to end users. This definition was recently updated to REASSURED, emphasizing essential elements that enhance diagnostic capability, i.e., Real-time connectivity and Ease of specimen collection [4]. This shift towards REASSURED criteria reflects a growing commitment to making diagnostics faster and more accessible, ultimately improving patient care in diverse healthcare environments.

To fulfil this task, leveraging technological growth provides various solutions. A recent trend is to integrate diagnostic platforms with artificial intelligence [5, 6], cloud computing [7, 8], and microfluidics [9, 10] to deliver high-quality POC tests for affordable and efficient healthcare management.

Among these, microfluidic devices play a crucial role, owing to the automation and miniaturization of complex and laboratory-based procedures onto compact lab-on-chip architectures. Fluid management is highly enhanced, with the possibility of easily manipulating liquid to deliver multiplexed analytical tools, with minimal sample consumption, while reducing analysis time.

Indeed, microfluidic channels can be configured to manipulate, process, and manage complex biological samples. These channels can be designed to obtain optimal size, flow rates, speed, and internal structures to (i) improve detection capability, (ii) deliver multiplexed platforms, and (iii) avoid external pumps.

Overall, these noticeable features facilitate user-friendly, rapid, and on-site detection platform fabrication, with the capability of reducing reagent and sample volumes, lowering test costs, and preserving materials, which is an essential point in resource-limited areas.

This role is also highlighted by the market trend study, revealing indeed microfluidics as a growing area for biomedical POC technologies. The global microfluidics market size is reported to grow from USD 40.25 billion in 2025 to USD 116.17 billion by 2034, with a compound annual growth rate (CAGR) of 12.50% during the forecast period from 2025 to 2034. This behavior is driven by the increasing use of microfluidics technology in point-of-care diagnostics, the development of diagnostic devices through technology, and the global rise in the prevalence of chronic illnesses [11].

Being characterized by miniaturization, portability, and low cost, sensing devices based on electrochemical techniques are highly indicated for integration with microfluidics. These combined systems, due to their inherent advantages, enable the development of effective POC detection platforms, featuring miniaturized size, automation of procedures, and user-friendliness [12–14].

Indeed, the equipment necessary for electrochemical signal acquisition is completely portable, enabling seamless integration with the aforementioned technological innovations cost-effectively [15, 16]. Consequently, electrochemical devices are especially well-suited for miniaturized analytical uses, and the integration with microfluidics facilitates lower sample usage and supports automated chemical reactions within small devices featuring microchannels [17].

Furthermore, the advances in new technologies, along with improvements in established microfabrication techniques, have created opportunities to utilize various materials for microfluidic device fabrication, such as paper, polydimethylsiloxane (PDMS), and double-sided adhesive coupled with polyethylene terephthalate (PET), in the development of these devices.

The scientific community has demonstrated significant interest in microfluidic electrochemical sensing platforms for application in several fields, resulting in an increasing number of reviews on this sector. These reviews cover a wide range of topics, dealing with different substrate materials, i.e., adhesive tape [18], paper [19–21] and PDMS [22], focusing on the fabrication processes [14], and emphasizing the role of modification with nanomaterials [23, 24], including mexene [25], nanozymes [26], and enzymes [27].

Additionally, most of these reviews discuss various types of sensors, including colorimetric, fluorimetric, and electrochemical [9, 10, 28–30].

Various sectors are usually covered [31–33] (namely, environmental monitoring [34], agrifood [35], wearable sensors [36–38], and drugs of abuse [39]), aiming at particular analytes detection, such as cancer biomarkers [40, 41], DNA and cellular analysis [42], B complex vitamins [43], and viral infection biomarkers [44].

This review stands out distinctly from the previous ones, focusing exclusively on electrochemical analysis, providing an in-depth discussion of wearable and point-of-care sensors, while microfluidic sensing devices not suited for analysis at the point-of-care/need are not covered. Moreover, this review is the first to include a comprehensive overview of applications for detecting target analytes in the most commonly used biological fluid. This includes sweat analysis for wearable sensors development, as well as blood, serum, saliva, and urine analysis for automating sensing procedures and detecting multiple analytes. The design, construction, and analytical performance of each microfluidic-based (bio)sensor are examined in detail to enhance understanding of their configuration and architecture. Additionally, the roles of paper, PDMS, and adhesive tape are examined, including the advantages and disadvantages of each material.

## Materials for microfluidics fabrication

Miniaturization of components and the avoidance of external pumps for fluid management are strictly mandatory to deliver an effective POC device based on a microfluidic electrochemical sensing platform. In this context, the use of the right substrate material is essential.

For this purpose, paper, PDMS, and adhesive tape with PET are the most exploited substrates in recent microfluidic device fabrication for integration with electrochemical detection, owing to their biocompatibility, flexibility, and ease of channel fabrication.

The inherent attributes of the paper make it an excellent substrate for developing microfluidics and integrating with electrochemical sensors [45, 46]. These characteristics comprise (i) the loading capability, offering a solution for the storage of reagents needed for the assay; (ii) the presence of a cellulose network, enabling fluid movement without external pumps, thanks to the capillary forces; (iii) the foldability, facilitating the creation of origami structures, and easily dealing with the shapes of the body when developing wearable sensors; and (iv) the easy integration with electrochemical electrodes [47, 48]. Finally, microchannel fabrication is straightforward, encompassing the use of wax printing to deliver hydrophobic barriers where fluid motion occurs. For these reasons, paper has been highly exploited for delivering microfluidic devices integrated with electrochemical sensors [49, 50].

Nevertheless, finding the right paper substrate according to the final purpose can be challenging. The use of bleaching substances and the final pH can affect the application of this substrate, as well as the flow capability, which is influenced by pore size and network cellulose. Additionally, the fabrication of precise channels is hindered by the wax-printing process, which does not allow for accurate control of hydrophobic barrier dimensions after the melting procedure.

Microfluidic devices for managing small volumes of fluids are also commonly made from PDMS polymer, due to its biocompatibility, flexibility, and cost-effectiveness [51]. For this substrate, to eliminate the need for external pumps for fluid motion, a particular foresight is needed by engineering capillary micropumps within channel networks. Compared to paper-based microfluidics, the fabrication procedure requires several steps, starting from the use of a mold which can be made of glass, silicon wafers coated with photoresist, or plastic materials like PET [52–54]. After several fabrication steps, a final stacking procedure is needed to adhere to the customized substrate/detection zone.

However, its ability to adhere to various substrates and the capability to mold it into microstructures enable PDMS application across tailored configurations. Additionally, its adhesion to connection pads typically does not interfere with the electrical signals, facilitating the integration of microfluidics with sensing modules.

While featuring the aforementioned characteristics, boosting the use of PDMS in microfluidic device development, its hydrophobicity is a severe issue. Indeed, this inherent feature can cause the absorption of hydrophobic small molecules, affecting the accuracy of the detection assay [55, 56]. Additional steps are therefore required, mainly based on (i) plasma exposure, although this procedure does not maintain the hydrophilicity for long times, (ii) UV modification, and (iii) chemical treatment. This last process should be carefully performed, taking into account that interaction with organic reagents easily creates PDMS swelling, leading to channel damage [57].

Considering this, it is crucial to carefully manage the leaching of uncured polymer and the distribution of hydrophobic molecules within the bulk of the polymer. This poses a limitation on the designs that can be created for biomicrofluidic systems utilizing PDMS.

Lastly, in recent years, the use of adhesive tape (coupled with PET as a supporting material) has emerged as a low-cost and easily accessible material for microfluidics development. Being commercially available in a variety of thicknesses, material compositions, and adhesive strengths [18], this substrate is suitable for inexpensive and rapid microfluidics fabrication, mainly based on the use of laser-engraving machines able to precisely create capillary channels and control their width [58, 59]. Stacking of layers can be easily accessed without complex bonding steps or chemical reagents, aligning the channels with the sensing zone, creating overlapping channels, inlets, and outlets.

Nevertheless, as a limitation to be considered, the degradation of the adhesive can affect the bonding strength, resulting in the device delamination and multilayer structure loss. For example, typical working temperature ranges between 15 °C and 35 °C, as extreme conditions lead to issues related to loss of stickiness and channel structure.

Additionally, channel width is intrinsically connected to the laser device, since the spot size of the laser determines the minimum channel dimensions.

Besides these commonly used materials, a few papers reported the use of other substrates which are not usually employed for microfluidics fabrication, including polyimide [60, 61], silicone [62, 63], and 3D-printed materials [64, 65], owing to their practical drawbacks, such as biocompatibility issues and poor flow capacity, eventually requiring an external pump or additional treatment.

## Application of microfluidic electrochemical devices for wearable sensors development: sweat analysis

Boosted by the rapid rise of technological advancements in terms of miniaturized electronics, wireless connectivity, and smart substrates, wearable sensors are gaining a primary role in real-time health monitoring, specifically applied for biomarker detection in sweat samples. Particularly, integration with microfluidic devices facilitates the collection of sweat during perspiration and delivering it to the right area, either the reaction or sensing zone, by fluidic channels and/or capillary micropumps, without the need for an external pump.

### Paper-based microfluidics

The distinctive features of the paper discussed earlier render this substrate highly well-suited for the development of microfluidic systems used in electrochemical wearable sensors. The smooth and efficient flow of biofluids, driven by capillary forces, enables multiple configurations. This includes designs such as lateral flow and vertical arrangements, inspired by origami structures, allowing for enhanced functionality and adaptability in wearable technology.

Lateral flow is a well-established approach in sweat management finalized for target analyte detection using wearable sensors. Exploiting paper wicking capability, the sweat biofluid is first sampled and then capillary forces in the cellulose network microchannels are exploited to direct the sampled fluid to the sensing zone.

As an example, recently, Fiore et al. designed a wearable microfluidic platform to perform a competitive magneto-immunoassay for cortisol detection in sweat, integrating the system with a plastic screen-printed electrode (SPE) and a near-field communication wireless module [66]. In this study, the cellulose network not only enabled the sweat collection and flow to the reaction zone but was also used as a reagent loading to perform the various immunoassay steps in a semi-autonomous way. The developed device enabled the amperometric detection of cortisol, exploiting (i) the loading of magnetic beads functionalized with monoclonal antibodies for the recognition of cortisol, and (ii) the acetylcholinesterase enzyme to give an amperometric response inversely proportional to the target cortisol, after the addition of the enzymatic substrate. The reliability of the developed analytical platform was demonstrated by the quantification of the cortisol level in volunteer sweat during cycling activity.

**Fig. 1:**
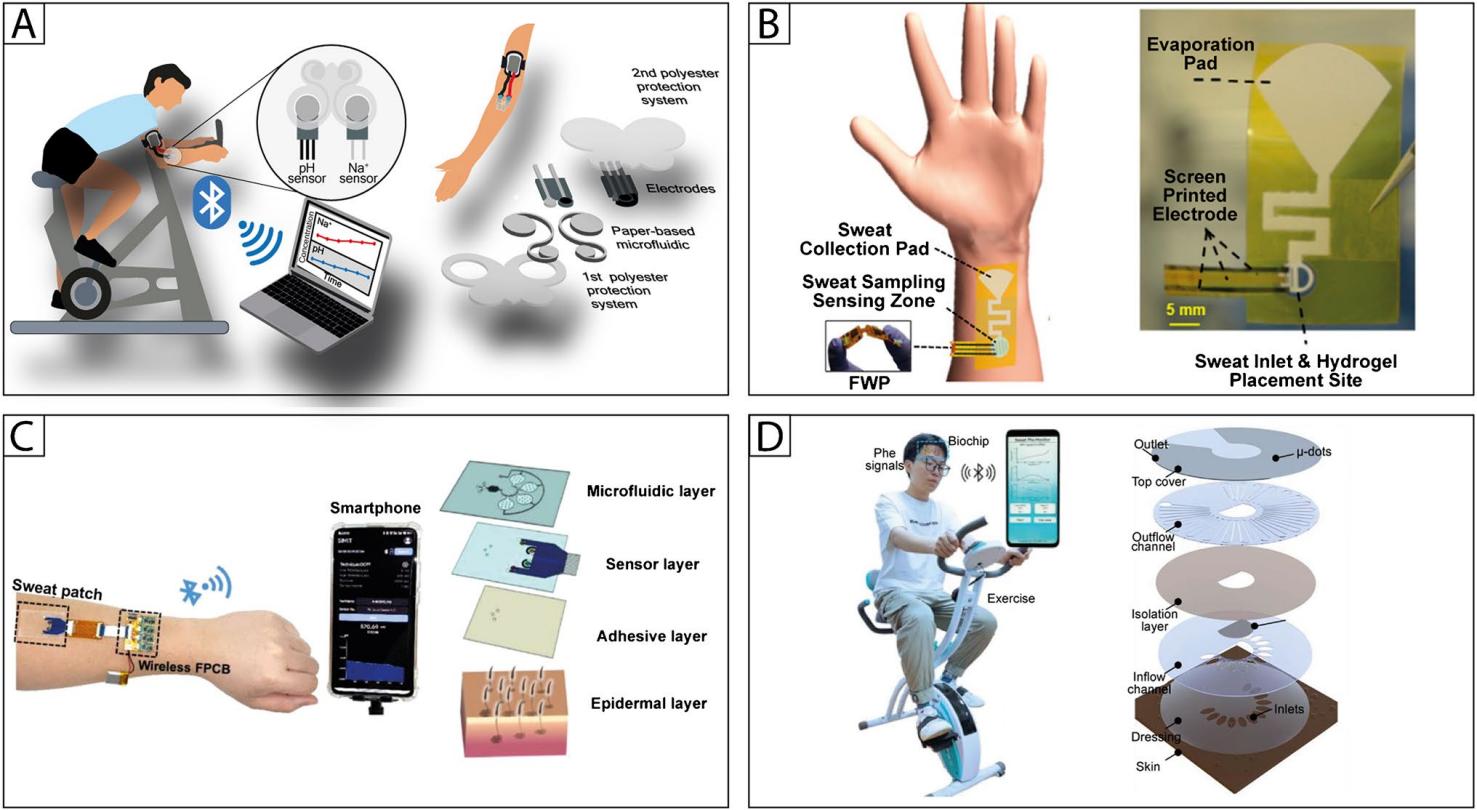
**A** Wearable electrochemical device based on butterfly-like paper-based microfluidics for pH and Na^+ ^monitoring in sweat [67]. **B** Wearable electrochemical sensing platform coupled with paper-based sweat extraction for continuous lactate monitoring [68].** C** Sequential microfluidic multi-sensing patch for biofluid sampling and sweat analysis [84].** D** Sweat phenylalanine multimodal analytical biochip for tracking exercise metabolism [59]

To manage the important problem of mixing of the newly secreted sweat with the already sampled and analyzed one, the same authors reported a microfluidic device designed with a butterfly-like configuration, integrating two ad hoc modified screen-printed electrodes for pH and Na^+^ monitoring during cycling activity [67] (Fig. 1A). The lateral flow configuration uses a Scottex® tissue paper layer having two circular microfluidic sampling zones, acting as a sweat collector, and the two SPEs allocated above them. To enable the target identification, the two SPEs were ad hoc modified with iridium oxide and an ion-selective membrane for the potentiometric detection of pH and Na^+^, respectively. This sensing area was then connected by the butterfly channels to a waste zone for sweat discharge.

To perform sweat extraction at rest without the need for exercise or chemical stimulation, Saha et al. coupled the paper microfluidic device with a hydrogel-containing pad [68] (Fig. 1B). While the hydrogel facilitates sweat extraction through an osmotic pumping mechanism, the paper microfluidic channels transport it to an evaporation pad via capillary forces. Below the osmotic hydrogel pad, electrodes screen-printed on polyimide provide real-time lactate monitoring by amperometric detection, exploiting lactate oxidase previously immobilized onto the working electrode surface.

Applying this system coupled with a custom flexible wearable wireless potentiostat, long-term sensing capability from fresh sweat were obtained, i.e., 2h.

Besides lateral flow configuration, the use of vertical microfluidics utilizing origami configuration in electrochemical paper-based devices represents a well-explored approach in the recent literature. Indeed, the foldability of paper facilitates the fabrication of this configuration and its application for continuous analysis of target analytes in sweat. This is primarily achieved through a multilayered architecture where (i) a first layer in direct contact with the skin absorbs sweat, (ii) a sandwiched layer containing miniaturized screen-printed electrodes detects the target analyte, and (iii) a final evaporation layer allows sweat to evaporate. This configuration ensures that the fresh liquid flows into the detection zone, while the already used sweat flows away and evaporates from the last layer, thus avoiding sweat accumulation in the device.

Following these concepts, four research groups reported a similar multi-squared layer configuration able to ensure the flow of sweat from the sampling zone, through the sensing zone, to the evaporation area. Hydrophobic barriers on Whatman chromatographic paper were fabricated using wax, with desired shapes and sizes, while the electrodes were directly screen printed on paper and modified ad hoc for (i) glucose [69], glucose and lactate [70], and glucose, creatinine, uric acid amperometric detection [71], and (ii) sodium and potassium potentiometric detection [72]. Integration with custom-fabricated printed circuit boards (PCBs) enables the accurate detection of the target analytes during physical activity.

### PDMS-based microfluidics

Microfluidic devices made from PDMS are highly advantageous for wearable technology owing to their skin-like properties (such as softness, flexibility, and skin compatibility).

To avoid the use of mechanical pump machines for sweat collection during perspiration, a combination of capillarity and the natural pressure of the eccrine glands must be exploited. To obtain this, the width of the fluidic channels must be properly and carefully assessed.

With the aim of avoiding the mixing of the sweat newly secreted with the already sampled and analyzed one, eventually causing interference, the structuration of a precise microfluidic is mandatory. To accomplish this, the usual approach is to merge inlet channels with a sensing area and a final outlet channel/reservoir, being able to remove from the sensing zone the analyzed fluid [73–76].

Following these principles, Sun et al. reported two separate microfluidic channels manufactured by an automated mechanical cutting machine, located in the same PDMS substrate, for the detection of glucose and lactate [77]. Each one of the two devices comprises four inlet ports connected to a reservoir where the amperometric detection of the analytes takes place by screen-printed electrodes modified with the proper enzyme. An outlet channel ensures the discharge of the old sweat, enabling the freshly produced one to enter the inlets to refill the sampling reservoir.

Niu et al. fabricated a tree-like microfluidic chip with a specific channel configuration for each region, depending on the relative task [78]. In detail, a root-like collection structure, a trunk-like detection region, and a leaf-like discharge structure were carefully designed and combined with (i) a multiparametric electrochemical sensor for the potentiometric monitoring of K^+^, Na^+^, and amperometric glucose and lactate detection during physical activity and (ii) a microheater utilized to enhance the sweating rate and maintain temperature stability in the detection area. The results demonstrated accurate detection within the physiological concentration range of the target analytes in sweat (namely, 1–32 mM K^+^, 10–160 mM Na^+^, 0–250 μM glucose, 0–20 mM lactate) during stationary bike activity as aerobic exercise.

To enhance sample collection efficiency, Xu et al. introduce capillary pumps between the inlet and outlet channels, above the sensing electrodes, to effectively absorb sweat [79]. This ensures that the detection area always contains fresh sweat, with a fast discharge of the liquid from the outlet. Using this approach, the authors simultaneously monitored glucose, K^+^, and Na^+^ during stationary running activity, integrating the microfluidic/sensing device with a multiparameter detection circuit and a Bluetooth module.

With the same task to achieve an optimal sweat flow in the microfluidic chip, Zhao et al. integrated the usual configuration (namely, inlet, sensing zone, and outlets) with a hydrogel film to assist the drawing of sweat, without the need for exercise to obtain perspiration [80]. The sensing tool was successfully applied for the monitoring of cortisol, Mg^2+^, and pH during 12 h, under several conditions, including oral supplements, intense exercise, and stressful state.

A supplementary aim could be facilitating the delivery of the sweat to different detection zones to accomplish multiple detection of analytes with different electrodes or different techniques.

For the first purpose, liquid PDMS was deposited directly onto filter paper substrate, designing microfluidic channels having an inlet, three channels, and three assay chambers [81].

Here, glucose and lactate detection were achieved by the amperometric signals generated from enzyme-catalyzed reactions with the analytes at Prussian blue/Au/SPE, while Cl^−^ potentiometric detection was performed using ion-selective-modified SPE. A linear response range covering the normal levels of the three target analytes in human sweat was obtained, with a limit of detection equal to 0.47 mM, 4.8 μM, and 2.9 mM for lactate, glucose, and Cl^−^, respectively.

Coupling different detection techniques is a valuable strategy to enhance the sensing reliability. To integrate the amperometric detection of glucose and lactate with the colorimetric assay for chloride and pH, Rogers’ research group designed a circular-shaped microfluidic device [82]. The device is engineered to drive the sweat from the sampling area located in the outer crown of the miniaturized system, through the colorimetric sensing zone, to the central electrochemical sensing zone. The entire sensing tool is successfully used during a stationary biking activity, being coupled with a miniaturized chip equipped with NFC functionality for smartphone wireless data extraction.

Iontophoresis provides a highly effective method for cases where the target analyte is not intrinsically related to physical activity. Utilizing microfluidics to drive the induced sweat from the collecting zone to the sensing area is an efficient approach to perform the sweat analysis autonomously [83–86].

As example, to drive the iontophoretic-induced sweat to four detection zones, Sun et al. proposed a microfluidic device based on capillary retention valves, pumps, and bursting valves, to (i) prevent sweat from backflow, (ii) improve the flow rate, and (iii) sequentially fill in the collecting/sensing chambers [84] (Fig. 1C). The sensing tool was applied for sweat analyses in a sedentary state, to monitor the concentrations of Na^+^, pH, glucose, and ethanol. The detection was achieved exploiting ion-selective SPEs for potentiometric Na^+^ and pH detection, while enzyme-modified SPEs were used for amperometric detection of glucose and ethanol.

In another work, Wang and Sempionatto research group exploited the use of microfluidics to drive the sweat, stimulated by iontophoresis coupled with pilocarpine usage, from three sampling zones to the detection area [85]. As proof of concept, the glucose detection was obtained by glucose oxidase–modified screen-printed electrodes over microfabricated gold collectors.

### Adhesive tape and PET

As an alternative to well-known paper and PDMS materials, recently, the use of adhesive tape is gaining high consideration, owing to its widespread commercialization and easy customization by laser engraving. A valuable and well-exploited approach is to couple double adhesive tapes with a transparent plastic support, typically PET, usually happening through a vertical multilayer integration of the various substrates. The fabrication of the desired microchannels in the adhesive tape is usually performed through a laser machine cutter/engraver.

Being the mixing of old and newly sampled sweat an event that must be highly limited, several approaches have used adhesive tape coupled with PET to address this issue.

Following these concepts, Liao et al. designed a biomimetic microfluidic sweat collection patch [58] encompassing four layers based on adhesive tape and PET. These include inlet and outlet holes, wedge-shaped channels, a detection chamber, where electrodes are screen-printed on PET, and discharge channels. The developed system enables rapid transport to the detection chamber by dynamically changing capillary forces in the bionic wedge-patterned channel. After the target analyte detection (i.e., glucose and lactate, using glucose oxidase– and lactate oxidase–modified Prussian blue/AuNPs SPEs, respectively), the sweat flows out through the discharge channel, effectively avoiding the mixing of old and new sweat. The sensing platform is succesfully applied to analyze changes in glucose concentration at different dietary intakes and changes in lactate metabolism after exercise.

**Fig. 2:**
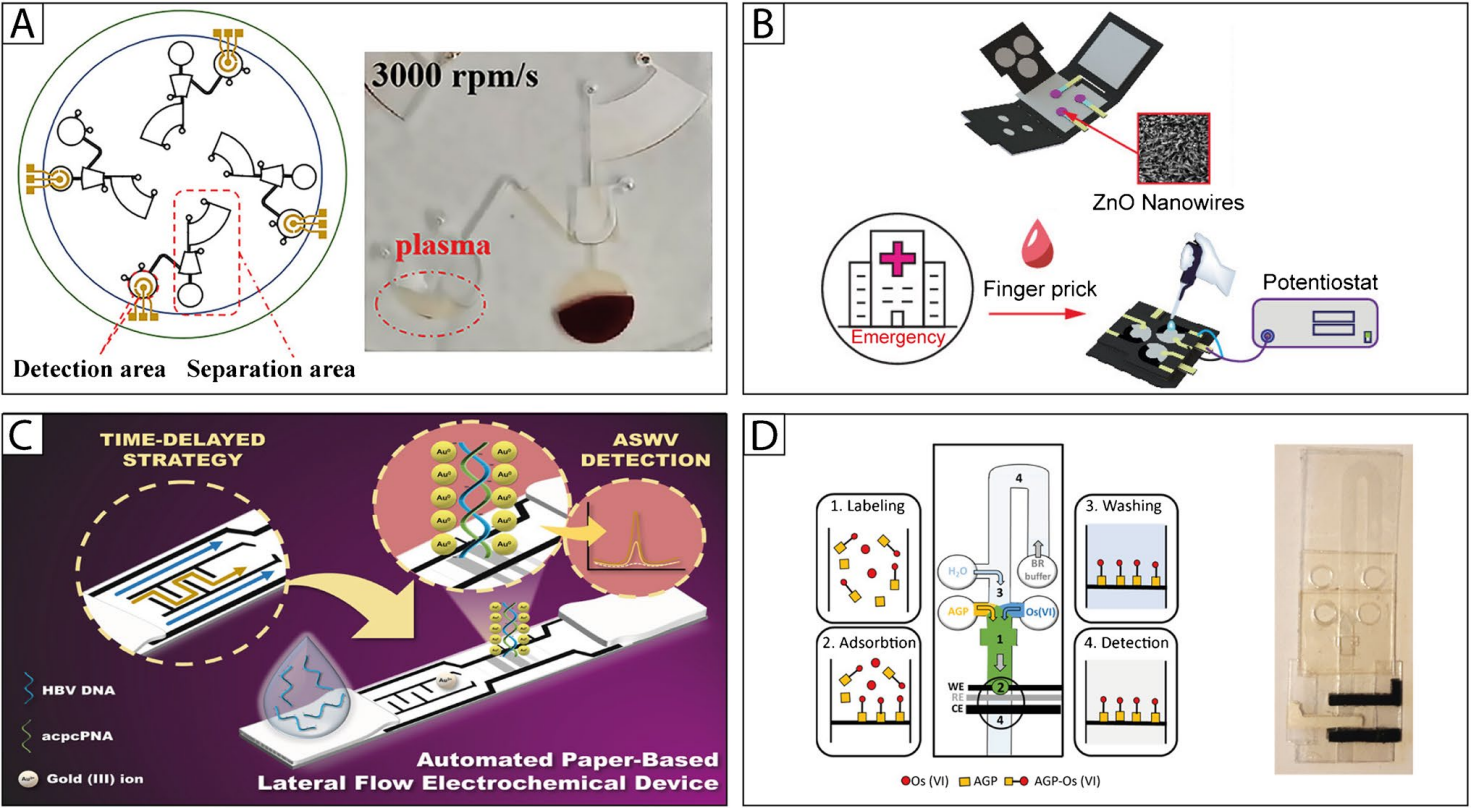
**A** Detection of VEGF165 in whole blood based on a centrifugal microfluidic chip [90]. **B** Paper-based origami biosensor for point-of-care detection of cardiac protein markers in whole blood [96]. **C** DNA sensor for the detection of the hepatitis B virus using an automated paper-based lateral flow electrochemical device [102]. D Pump-free microfluidic device for the electrochemical detection of α1‑acid glycoprotein [108]

As a multipurpose microfluidic module for a wearable sweat phenylalanine sensor, Zhong et al. reported the fabrication process involving the patterning of inlets and chambers on multilayered PET-adhesive tape-PI substrates [59] (Fig. 2D). This device allowed for fast sweat collection, discharging and refreshing, integrating with the visualization of serpentine outflow channels for sweat loss status assessment. The microfluidic sensing device enabled the voltammetric detection of phenylalanine using molecularly imprinted polymer (MIP)-SPE, providing physiological insights into health and metabolism after protein intake during exercise.

Considerable studies have been made by Emaminejad’s research group for the development of wearable sensors integrated with electronic readout systems [87–89].

As a first basic approach based on double-sided tape interlayered in PET, the Emaminejad group created a sweat collection chamber combined with a sandwiched-electrode array structure for the amperometric detection of glucose [87]. Here, the fluid motion is induced by a temperature gradient profile, which interacts with an electric field, both induced by electrode actuation. Upon iontophoretic stimulation, sweat is sampled by the microfluidic device, worn by the volunteer, and analyzed by amperometric detection using the glucose oxidase-modified Prussian blue-gold-patterned electrode array system integrated with an electronic device.

As the complexity of the microfluidic system increased, a high-technology “lab-on-body” platform was developed to provide complete automatic biofluid management [88], which encompasses (i) a double-sided skin-adhesive film, (ii) a biochemical sensing electrode array patterned on PET and modified with glucose and lactate oxidase, (iii) a double-sided tape and PET serving as the microfluidic layer for sweat sampling, flowing, and compartmentalization, (iv) a thermo-responsive hydrogel array layer that functions as a valve, (v) a microheater electrode array for valve switching, and (vi) pressure regulator embodiments. The entire miniaturized and fully automatic system was utilized for glucose and lactate monitoring during physical activity by connecting to a PCB module wirelessly linked to a receiving smartwatch.

The same research group lastly reported a freestanding electrochemical sensing system that combines the four main components (namely, microfluidics, sensors, electronics, and output), all integrated into a self-developed smartwatch [89]. The microfluidics, based on double-sided tape attached to PET and featuring outlet ports, was assembled with noble metal-based electrode arrays and housed in a 3D-printed case that includes a PCB module and an output display for data visualization. Utilizing the developed analytical platform, iontophoretically induced sweat was analyzed for glucose detection before and after lunch, while lactate detection occurred during running exercise. In both cases, the amperometric detection aligned with the physiological ranges of the target analytes.

## Application of microfluidics for biomarker detection in blood

Clinician requirements focus on a minimally invasive sampling procedure to ensure minimal pain for patients. An approach based on microfluidic electrochemical–based sensors can allow for the use of low sample volume, enabling minimally invasive sampling. Additionally, considering the complexity of this matrix, an effective yet straightforward separation of plasma from cellular components (namely, red/white blood cells and platelets) may be required, depending on the final application. In traditional methods, this step is typically performed by a first centrifugation procedure with laboratory-based instrumentations. The use of microfluidics can allow for the scaling and miniaturization of this separation procedure, along with requiring minimal blood volume.

For example, He et al. developed a centrifugal microfluidic chip integrating a separation and a sensing unit [90] (Fig. 2A). The microfluidic platform is fabricated with PDMS and is designed to allow for (i) a 5-min plasma-hemocytes separation step, happening through centrifugal rotation, and (ii) the detection of vascular endothelial growth factor 165 (VEGF165). The plasma is collected into a detection chamber where a sandwich-type sensing strategy is applied for the final VEGF165 quantification by differential pulse voltammetry at the Au sputtered working electrode.

Farrokhnia et al. designed a planar microfluidic device coupled with a MIP-based electrochemical sensor for succinate detection in blood [91]. In this case, the microfluidic channels are structured with pressure-sensitive adhesive, while the plasma separation occurs through a customized filtration membrane. Using 120 µL of blood sample, the succinate detection is accomplished in 25–30 min, achieving a detection Limit of 5 nM, in the Linear range of 50 nM–250 μM.

To perform plasma-red blood cell separation, commercial membranes were also applied by developing multilayer microfluidic paper–based devices based on lateral flow or vertical flow configurations.

Keyvani et al. exploited the presence of a capillary micropump and parallel capillaries in a PDMS microfluidic device for autonomous flowing of fluid through a commercial filter membrane (Sterlitech) [92]. The filtered plasma is then delivered to a separate PDMS chamber covering the SPE for the detection of the circulating DNA of human papillomavirus (HPV) by DPV. Specifically, to perform the assay, the graphene oxide-modified SPE was grafted with a complementary DNA strand, specific to the target HPV-DNA, and the highly sensitive Ru(NH_3_)_6_^3+^  + Fe(CN)_6_^3−^ electrochemical system was employed for the HPV-DNA detection.

Gautam et al. designed a dumbbell-shaped microfluidic channel made of a combination of LF1 membrane and Whatman grade 1 for plasma separation and detection zone arrangement, respectively [93]. This configuration allowed for a consistent flow of filtered plasma from the two external zones (LF1 membrane) of the dumbbell to the central detection zone (Whatman grade 1) for the final cyclic voltammetric detection of ascorbic acid by SPE in a total volume of 180 µL of whole blood samples.

Owing to its reliability and cost-effectiveness, the separation membrane from Pall Corporation has frequently been integrated into paper-based microfluidics, exploiting lateral flow and origami configurations.

The blood separation membrane (Vivid GX Membrane, Pall Corporation) was coupled with the fluidic capability of nitrocellulose paper and double-sided adhesive microchannels to deliver a multilayered electrochemical device for antibodies anti-SARS-CoV-2 nucleocapsid protein [94]. This smart architecture enabled the chronoamperometric detection of the target analyte using a low volume of blood (namely 10 µL) and 20 min as total reaction time. For this purpose, an electrochemical immunoassay was automatically performed exploiting the microfluidic device, while chronoamperometric detection happened at the stencil-printed carbon electrode.

Conversely, a vertical flow strategy was applied to embed the plasma membrane in a first layer of an origami paper-based configuration [95]. A second paper layer, comprising wax-based channels, was used to deliver the plasma to the third layer comprising two SPEs on the same Whatman layer for the detection of C-reactive protein (CRP) and prealbumin (PAB). Exploiting anti-CRP aptamer-functionalized SPE and anti-PAB antibody-functionalized SPE, DPV measurements enabled CRP and PAB detection with linearity up to 1 µg/mL for both biomarkers, while a LOD equal to 5 and 10 pg/mL for CRP and PAB, respectively.

**Fig. 3:**
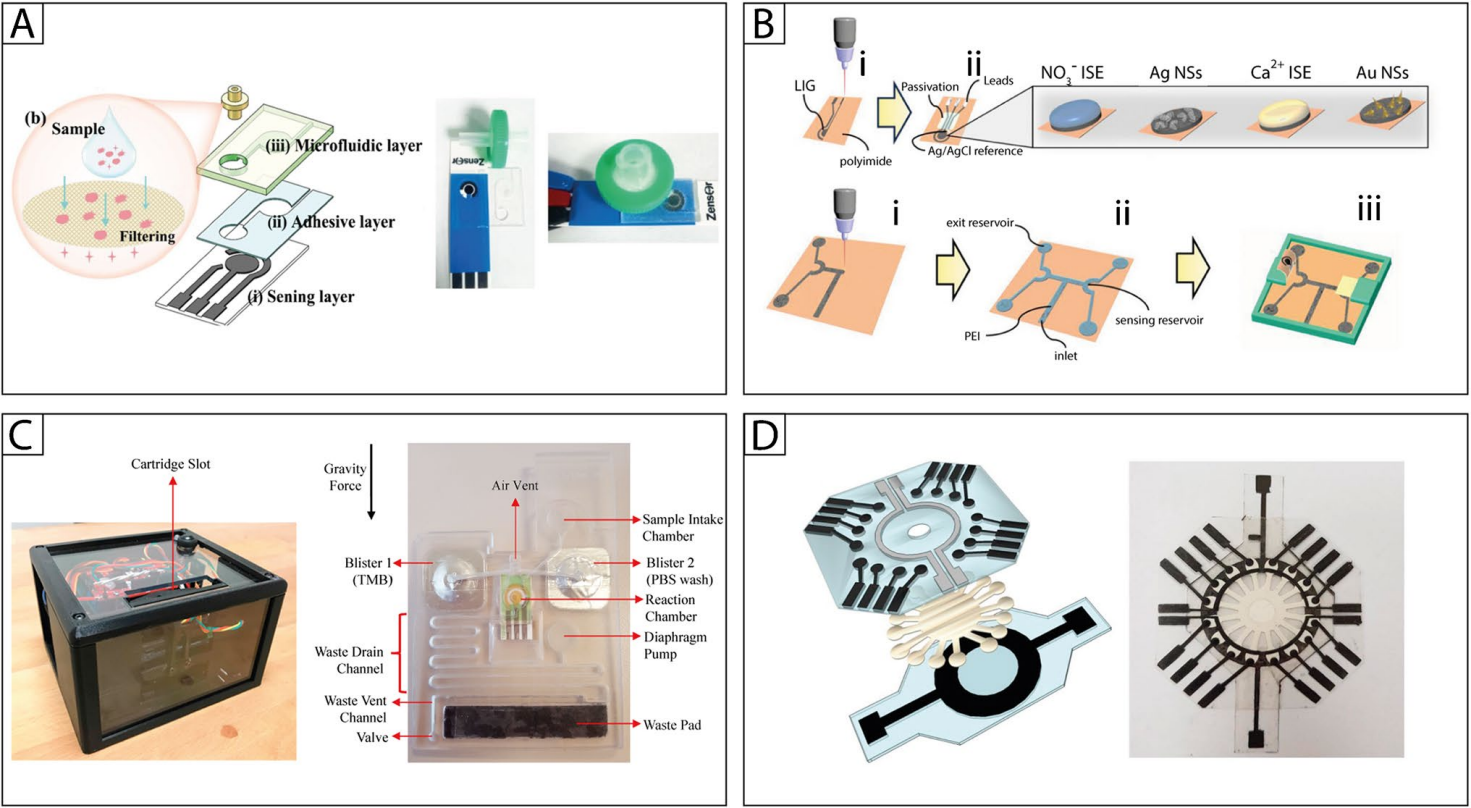
**A** Nanozyme-based electrochemical sensor integrated with a microfluidic cell for on-site uric acid detection [111].** B** Laser-induced graphene microfluidics integrated sensors for on-site biomedical monitoring [60].** C** Fully automated electrochemical point-of-care biosensor for hCG detection in human urine samples [114].** D** Electrochemical paper-based microfluidic device for multiplexed analysis of urine samples [115]

Using a similar origami approach, Fu et al. designed a foldable/detachable multilayer system for multiplexed detection of three cardiac proteins in whole blood [96] (Fig. 3B). By flapping and detaching the several layers containing (i) a plasma separation membrane, (ii) an absorption paper, and (iii) a signal readout flap, the system allows for the simultaneous detection of cardiac troponin I, brain natriuretic peptide-32, and d-dimer using electrochemical impedance spectroscopy, in a Low volume of 20 µL of finger blood sample.

Using the same separative membrane, Caratelli et al. employed a vertical flow configuration to develop a three-layer paper setup for the analysis. The device consists of a first office paper for SPE printing, a second filter paper to preload the substrate for the enzymatic reaction with butyrylcholinesterase, and a third plasma separation membrane for blood filtration [97]. Inhibition activities of physostigmine, rivastigmine, and donepezil were measured in blood samples, showing linearity up to 0.5 μM, 25 μM, and 30 μM, with detection limits of 0.009 μM, 0.4 μM, and 0.3 μM, respectively.

With the aim of incorporating the sampling system [98] or to automate the entire detection procedure [99], two noteworthy approaches applied microfluidics to reach their purpose. In the first case, hollow microneedles were exploited to collect the blood sample, and a PDMS microfluidic system was integrated with a sensing device to detect glucose in blood, as a potential wearable system [98]. The device is designed to extract the biofluid from the skin using negative pressure by a vacuum suction button on the microfluidic platform and to deliver the blood to the sensing area. Here, glucose is detected by chronoamperometry at the glucose oxidase-functionalized SPE.

In order to achieve automation of magneto-assay, Prat-Trunas et al. combined paper-based microfluidics and electrochemical detection in a 3D-printed holder for lactate dehydrogenase detection in whole blood [99]. The paper microfluidics architecture exploits a reservoir area, a sensing zone, and an absorbent pad to manage the various steps of the assay, including washing, flowing, and sensing.

## Application of microfluidics for biomarker detection in serum

Owing to the relatively easier management of serum compared to whole blood, applying microfluidic devices for the electrochemical detection of analyte(s) allows multiple functions.

Automation of experimental procedures, harnessing paper as a substrate for microfluidic fabrication, is a convenient solution for managing the various steps of the analytical assay. The foldability and easy patterning of the paper substrate are indeed essential to deliver the final device by creating the needed microfluidic channels or specifically shaped areas.

With the aim of facilitating the assay procedures, Qi et al. reported a notable paper-based microfluidic device able to assist in the synthesis of molecularly imprinted polymer and to perform the assay [100]. The highly structured paper platform comprises four functional folding parts, including (i) working electrode part, (ii) one circular counter/reference electrode part for synthesis, (iii) one circular counter/reference electrode part for analyte detection, (iv) a movable valve part to direct the sample to the detecting zone, and (v) a washing channels part. The proposed device is applied for the detection of carcinoembryonic antigen as a model target for the analysis in serum, with a linearity up to 500.0 ng/mL, and a LOD equal to 0.32 ng/mL.

In order to remove the interference from the convective component of fluid motion, Yakoh et al. developed a noteworthy rotational and vertical flow immunosensor for α‑fetoprotein detection [101]. The target analyte quantification was performed by electrochemical impedance spectroscopy measurements at the electrode stencil-printed on the paper disk. To perform the immunoassay, the various steps of transfer, switch, and stop fluid flows needed for the assay can be performed by manually rotating the paper disk. As a result, the authors were able to enhance the analytical performance of the sensor, i.e., by lowering the LOD from 1.65 pg/mL to 3.54 fg/mL.

Based on the well-known lateral flow strategy and exploiting wax patterning of nitrocellulose paper, Srisomwat et al. created an innovative automated device for detecting hepatitis B DNA [102] (Fig. 3C). The device features wax barriers and straight channels that guide a gold (Au) probe and the DNA target to a peptide nucleic acid (PNA) capturing agent in the detection area. The authors designed slowing barriers, which allow the Au probe to arrive at the detection area once the DNA hybridizes with the PNA capturing agent. Then, stripping voltammetry is used to detect the gold bond to the DNA-PNA complex. This method allows for the measurement of target DNA in less than 7 min, with high specificity and minimal background noise.

Performing multiple analyte detection is a valuable approach to reduce sample consumption and increase the functionality of the sensing device. With the aim of directing the serum sample to two different detection zones, two similar approaches exploited Whatman Grade 1 filter paper to reach the goal. In detail, Ong et al. developed a two-way microfluidic device to detect conjugated and unconjugated bilirubin simultaneously [103]. To distinguish between the two bilirubin forms, one of the two microfluidic channels (fabricated with glass fiber pads) was loaded with caffeine sodium benzoate to release the albumin-bound bilirubin. The subsequent square wave voltammetric detection was achieved at the laser-induced graphene electrodes.

Wang et al. employed wax-patterned channels for the capillary-driven flow to deliver the serum to two ad hoc modified screen-printed working electrodes for the aptamer-based detection of carcinoembryonic antigen and neuron-specific enolase [104]. The multilayered platform was able to detect the target analytes in serum samples, resulting in a good correlation with electrochemical luminescence (ECL) equipment.

To accomplish a similar task, Boonkaew et al. in their study introduced an improved paper analytical immunosensing device with dual flow channels to simultaneously detect hepatitis B virus (HBsAg) and hepatitis C virus core antigen (HCVcAg) [105]. The device automates washing and reagent steps of the immunological assay multilayered configuration coupling filter papers with transparency films and double-sided adhesive tape. It shows excellent sensitivity with detection limits of 18.2 pg/mL for HBsAg and 1.19 pg/mL for HCVcAg.

To enlarge the reliability of the sensing platform, Escarpa research group reported a double technique detection microfluidic platform for the assessment of transferrin saturation in serum samples [106]. The detection is achieved by the ratio between transferrin-bound iron, assessed by the colorimetric approach, and total iron-binding capacity, evaluated by the electrochemical approach. In detail, the microfluidic paper–based device was designed to integrate both colorimetric and electrochemical detection reservoirs, communicating via a microchannel acting as a chemical reactor, and with preloaded reagents. The dual-detection platform was then successfully applied for the analysis of serum samples from ischemic stroke patients, showing excellent accuracy in comparison with the reference method.

Alongside the paper substrate, four different strategies exploited double-sided tape and plastic substrates to deliver electrochemical point-of-care microfluidic devices.

Recently, exploiting laser technology, Chen et al. created a sensing device embedding in the same polyimide substrate the laser-induced graphene (LIG) microfluidic channels and the electrode, for simultaneous quantification of dual targets, namely dopamine and uric acid [61]. The created hydrophilic graphene microfluidic channel allows for the easy flow of the sample from a central inlet port to the sensing areas where electrodes are located. Dopamine and uric acid are successfully detected by differential pulse voltammetry, obtaining satisfactory recovery in human serum samples.

Integrating (i) multilayered laser-cut double-sided tape to fabricate the microchannels and (ii) micropillar array electrodes on poly(methyl methacrylate) (PMMA) substrate, Chen et al. designed a multilayered microfluidic device for glucose, uric acid, and sarcosine in human serum [107]. In this case, the solution flows through the microchannels to three different sensing areas thanks to a negative pressure generated by a manual syringe.

To accomplish the several tasks for the detection of the target analyte, namely α1-acid glycoprotein, the Henry group designed a pump-based microfluidic system to assist the electrochemical detection of α1-acid glycoprotein (AGP) [108] (Fig. 2D). The needed reagents were added one by one in separate reservoirs/inlets and mixed by flowing in the main channel, thanks to absorbent paper acting as a capillary pump. Specifically, during the capillary-driven flow, the AGP was mixed with Os (VI) (serving as an electrochemical tag). The formed complex was then delivered to the sensing area for the electrochemical detection at the stencil-printed electrode.

The microfluidic device exhibited a linear range up to 2000 mg/L (*R*^2^ = 0.990), with a limit of detection equal to 231 mg/L. Commercial serum samples were analyzed to demonstrate the success of the method.

Coupling capillary micropumps within a PDMS substrate is a valuable method for utilizing this material in microfluidics, eliminating the need for external electrical pumps. Following this approach, Xu et al. designed a PDMS microfluidic system that integrates an Ni_2_P-modified SPE in the detection chamber and a NaOH-loaded circular filter paper inside the microchip inlet for the non-enzymatic detection of glucose [109]. The PDMS layer mainly comprises an inlet, a zigzag microchannel, a detection chamber, an elliptical micropost array-based capillary pump, and a final outlet. The analytical platform showed two linear detection ranges (1 μM–1 mM and 1–6 mM), with a detection limit of 0.32 μM.

## Application of microfluid-based sensing tools in various biofluids: saliva and urine

### Saliva

Owing to the practicality of saliva sampling, it is potentially a good candidate for developing point-of-care analytical tools, as the collection of this biofluid remains quite easy. In this case, the microfluidic approach helps in the automation of the sensing procedure and in adding filtering pretreatment.

As an example, a magneto-immunoassay was developed for the multiplexed detection of interleukin-8, tumor necrosis factor-α, and myeloperoxidase biomarkers in sputum [110]. The authors integrated a fluidic channel wax-patterned on paper, enabling the sequential addition of the magnetic nanoparticles, after external sample pretreatment and washing steps. The channels are connected to five electrochemical cells, and the whole system is then inserted in a custom-designed PMMA cartridge and connected to a custom-fabricated electronic component for the final readout. The analysis of sputum samples of healthy individuals and chronic obstructive pulmonary patients revealed statistically significant biomarker concentration differences between the two studied groups.

On the other side, with the goal of conducting the sample pretreatment and target detection on a single integrated platform, Zhao et al. [111] and Vinoth et al. [112] combined an electrochemical printed sensor with microfluidic devices that integrate filtering membranes. In the first instance, a 3D-printed PET microfluidic device features an inlet channel, an outlet channel, and a detection cell positioned above a nanozyme-based electrochemical printed sensor for uric acid detection (Fig. 3A). The sample is injected through a filtering device with a syringe into the initial inlet channel. In contrast, the second instance describes a fully autonomous PDMS microfluidic system that includes a custom porous filtering membrane at the inlet channels and horizontal capillary channels for sample flow. Once inserted into the device, the fluid is then directed to four sensing areas for the analyte detection. Using this setup, the simultaneous electrochemical detection of glucose, lactate, cholesterol, and uric acid from saliva samples occurs.

As microfluidics based on paper substrates can be easily tailored for a particular aim, this substrate was exploited to deliver a salivary thiocyanate detection system based on colorimetric (qualitative assay) and electrochemical techniques (qualitative detection), using low sample volume (20 μL) [113]. The wax-patterned paper was folded and wrapped around the back of the screen-printed electrode device for the electrochemical detection zone. A hollow capillary fabricated by a laser-engraving machine enables the flow to the colorimetric area, where the colorimetric assay takes place. A remarkably wide dynamic range is obtained for thiocyanate between 0.025 and 100 mM using the two sensing modes.

Laser engraving is a powerful technique for microfluidic fabrication; nevertheless, owing to the hydrophobicity of the starting substrate, namely polyimide, it creates several issues in fluidic channel delivery. Johnson et al. addressed this drawback by a hydrophilic treatment with polyethyleneimine using the 1-ethyl-3-(3-dimethylaminopropyl)carbodiimide (EDC) chemistry [60] (Fig. 3B). Using this strategy, the authors fabricated a multiple sensing device for uric acid and calcium detection, exploiting laser-induced graphene electrode modified with gold nanostructures and ion-selective membrane, respectively.

### Urine

Urine test is a well-established biofluid in pregnancy tests, mainly using lateral flow semi-quantitative assays, and in detection of biomarkers associated with kidney function, i.e., creatinine and uric acid.

As application in the pregnancy test field, Yuksel et al. reported a fully automated system comprising (i) a disposable cartridge containing a sensor, reagents, and microfluidics, produced to process the sample and to automate all assay preparation steps and (ii) a portable analyzer, housing the electronics required to control the liquid flow throughout the cartridge and measure the signal [114] (Fig. 3C). The analytical system was applied for the detection of human chorionic gonadotropin (hCG), as a precise and rapid early pregnancy test, using a sandwich immunological assay. Indeed, thanks to the automated agitation device, the surface reaction rate is significantly improved compared to routinely performed sandwich assays, and therefore, a rapid detection of very low concentrations was achieved, recording a Limit of detection of 2.96 mIU/mL, within established clinical hCG levels for early detection of pregnancy.

Fatibello-Filho and coworkers fabricated a multi-electrochemical platform, based on a radial paper microfluidics channel, reporting the studies in two works [115, 116]. An array of 16 working electrodes was used to quantify simultaneously glucose, creatinine, and uric acid [115], and glucose [116] in urine samples. In detail, a multilayer architecture comprises two polyester layers where working and reference (top layer) and counter (bottom layer) electrodes are screen-printed (Fig. 3D). The Whatman filter paper–based microfluidics is sandwiched between these two polyester layers, enabling the flow of the sample from the center of the wax-patterned substrate to the peripheral detection zones, thanks to capillary forces. Here, the analysis of the target analytes happens by the screen-printed electrodes, exploiting glucose oxidase, iron-complex formation, and carbon black for the detection of glucose, creatinine, and uric acid, respectively.

Filter paper was also exploited by Wang et al. for the electrochemical detection of lead in urine. In this case, paper assisted in the removal of interference from human albumin [117]. Indeed, by loading the paper substrate with (NH_4_)_2_SO_4_, albumin was effectively isolated and retained in the paper network, allowing for Pb detection by a gold-plated plastic working electrode. Anyway, before the electrochemical analysis, the urine samples were filtered with membrane filters and were acidified with 37% HCl. The final square voltammetric detection in urine samples was in good agreement with reference method analyses, namely atomic absorption spectrometry.

## General remarks and outlook

Over the past few years, microfluidic devices have significantly improved the field of analytical platforms based on electrochemical POC sensors, offering accessible, affordable, and decentralized solutions for the detection of biomarkers in various biological fluids. The principal features of microfluidics, including minimal reagent and sample volume use, fast response times, and automation of fluid management, allow for the creation of miniaturized, wearable, and multiplexed detection tools suitable for real-time analyses and use in areas with limited resources.

In detail, the application of microfluidic platforms for developing wearable sensors has rapidly grown in recent years, capitalizing on the use of flexible, lightweight yet reliable substrates, along with their integration with advanced reading devices (Table 1). Although paper-based microfluidics can efficiently collect sweat, thanks to paper wicking properties, and manage liquid flow, thanks to capillarity, they tend to be fragile, particularly during long-term usage in physical activity. To address this issue, the majority of works focus on fostering the use of PDMS substrates, owing to their enhanced robustness. Additionally, employing double-sided adhesive tape enables easy customization of the microfluidic configuration while maintaining higher robustness and flexibility.

**Table 1:** Microfluidic electrochemical devices for wearable sensors development: sweat analysis

Target biomarker	Sensor	Microfluidic substrate	Microfluidic configuration	Microfluidic function	Detection technique	Linear range/LOD	Ref
Cortisol	SPE on polyester	Cordenons filter paper	Lateral flow	Sampling, discharging, reagents loader, magnetic immunoassay performing	Amperometry	10–140 ng/mL/3 ng/mL	66
Na^+^ and pH	SPE on polyester	Scottex® tissue paper	Lateral flow	Sampling and discharging	Potentiometry	Na^+^: 10^–3^−1 M/--- pH: 4–7/---	67
Lactate	SPE on polyimide	Whatman filter Paper Grade 541	Lateral flow	Sampling, discharging and hydrogel placement site	Amperometry	0–15 mM/350 nM	68
Glucose	SPE on PET	Filter paper	Vertical flow	Collecting and evaporating	Amperometry	0–1.9 mM/5 µM	69
Na^+^ and K^+^	SPE on PET	﻿Whatman #4 filter paper	Vertical flow	Collecting and evaporating	Potentiometry	Na^+^: 8–128 mM/--- K^+^: 1–32 mM/---	72
Glucose and lactate	SPE on Whatman paper	Whatman chromatography paper #1	Vertical flow	Collecting and evaporating	Amperometry	Glucose: 0.08–1.25 mM/17.05 μM Lactate: 0.3–20.3 mM/3.73 μM	70
Creatinine, glucose, uric acid	Penetrating deposition electrode on Whatman paper	Whatman #4 filter paper	Vertical flow	Collecting and evaporating	Amperometry	Glucose: 0–1 mM/7.9 μM Uric acid: 0–0.5 mM/1.32 μM Creatinine: 0–500 μM/7 μM	71
Uric acid	SPE on polyimide	PDMS	Lateral flow	Sampling, directing to sensing zone, discharging	Cyclic voltammetry	6.25–1500 μM/---	73
Levodopa and uric acid	Scraped electrode on Ti and Pt sputtered polyimide	PDMS	Lateral flow	Sampling, directing to sensing zone, discharging	Differential pulse voltammetry	Levodopa: 0–80 and 80–500 μM/0.29 μM Uric acid: 0–500 μM/0.85 μM	74
K^+^, Na^+^, glucose	SPE on polyimide	PDMS	Lateral flow	Sampling, directing to sensing zone, discharging	K^+^ and Na^+^: potentiometry Glucose: amperometry	K^+^: 1–32 mM/--- Na^+^: 5–160 mM/--- Glucose: 0–400 μM/---	75
K^+^	SPE on polyimide	PDMS	Lateral flow	Sampling, directing to sensing zone, discharging	Potentiometry	K^+^: 1–32 mM/---	76
Glucose and lactate	Commercial SPE on PET	PDMS, PET and adhesive tape	Lateral flow	Sampling, directing to two sensing areas, discharging	Amperometry	Glucose: 0–160 mM/7.34 μM Lactate: 0–25 mM/1.24 mM	77
K^+^, Na^+^, glucose, and lactate	Au sputtered electrode on polyimide	PDMS	Lateral flow	Sampling, directing to sensing zone, discharging	K^+^ and Na^+^: potentiometry Glucose and lactate: amperometry	K^+^: 1–32 mM/--- Na^+^: 10–160 mM/--- Glucose: 0–250 µM/--- Lactate: 0–20 mM/---	78
K^+^, Na^+^, Glucose	SPE	PDMS	Lateral flow	Sampling, directing to sensing zone, discharging	K^+^ and Na^+^: potentiometry Glucose: amperometry	K^+^: 1–32 mM/--- Na^+^: 1–128 mM/--- Glucose: 50–1000 µM/2.48 µM	79
Cortisol, Mg^2+^, pH	SPE on PET	PDMS	Lateral flow	Sampling, directing to sensing zone, discharging, and hydrogel film allocation	Cortisol: amperometry Mg^2+^: potentiometry pH: potentiometry	Cortisol: 1 nM-10 µM/0.072 nM Mg^2+^: 1 µM-10 mM/--- pH: 4.5–6.5/---	80
Lactate, glucose, and Cl^−^	SPE on filter papers (BWD Filter Co.)	PDMS onto filter paper	Lateral flow	Sampling and directing to three sensing areas	Lactate and glucose : amperometry Cl^−^: potentiometry	Lactate: 0–20 mM/0.47 mM Glucose: 0–200 µM/4.8 μM Cl^−^: 10–160 mM/2.9 mM	81
Glucose and lactate	Electron-beam evaporated gold electrode on polyimide	PDMS	Lateral flow	Sampling and directing to different sensing areas	Amperometry	Glucose: 0–400 µM/--- Lactate: 0–20 mM/---	82
CRP, cholesterol, and K^+^	SPE on PET	PDMS	Lateral flow	Sampling and directing to sensing zone	CRP and cholesterol : differential pulse voltammetry K^+^: potentiometry	CRP: 0–100 ng mL^−1^/--- Cholesterol: 0–120 μM/--- K^+^: 10^–6^−1 M/---	83
Glucose, ethanol, pH, and Na^+^	Commercial SPE on PET	PDMS, PET and adhesive tape	Lateral flow	Sampling, directing to four sensing areas, discharging	Glucose and ethanol: amperometry pH and Na^+^: potentiometry	Glucose: 20–120 µM/--- Ethanol: 5–45 mM/--- pH: 4–8/--- Na^+^: 10–200 mM/---	84
Glucose, Ca^2+^, Na^+^, K^+^, pH	Stencil-printed electrode on PET	PDMS	Lateral flow	Sampling and discharging	Glucose: amperometry Ca^2+^, Na^+^, K^+^, pH: potentiometry	Glucose: 5 −320 µM/--- Ca^2+^: 0.1–25.6 mM/--- Na^+^: 1–128 mM/--- K^+^: 0.5–32 mM/--- pH: 4–10/---	86
Glucose	SPE over microfabricated gold collectors	PDMS	Lateral flow	Sampling from three sampling zones, directing to a single sensing areas, and discharging	Amperometry	---/---	85
Glucose and lactate	Commercial SPE	Double-sided adhesive and PET	Lateral flow	Sampling, directing to two sensing areas, and discharging	Amperometry	Glucose: 50–250 μM/0.550 μM (In artificial sweat) Lactate: 5–35 mM/0.652 mM (In artificial sweat)	58
Phenylalanine	Thermally evaporated Au electrode on PET	Double-sided adhesive and PET	Lateral flow	Sampling, directing to serpentine and working electrode, discharging	Differential pulse voltammetry	10–300 μM and 300–1000 μM/4.7 μM	59
Glucose	Evaporated Cr and Au electrode on PET	Double-sided adhesive and PET	Temperature induced flow	Sampling	Amperometry	100–600 µM/17.56 µM	87
Glucose and lactate	Evaporated Cr and Au electrode on PET	Double-sided adhesive and PET	Flow by thermo-responsive hydrogel valves	Sampling, directing to the sensing area, and discharging	Amperometry	Glucose: 50–400 μM/--- Lactate: 2–10 mM/---	88
Glucose and lactate	Noble metal electrode array patterned on adhesive tape	Double-sided adhesive and PET	Flow by sweat pressure and capillarity	Sampling, discharging	Amperometry	Glucose: 0–1000 μM/1.7 M Lactate: 0–20 mM/4.6 µM	89

*CRP* C-reactive protein, *PDMS* polydimethylsiloxane, *PET* polyethylene terephthalate, *SPE* screen-printed electrode

When dealing with the complex blood matrix, the use of microfluidic platforms is mainly devoted to accommodating separation membranes or to be directly involved in the separation process (Table 2). In this context, commercial separation membranes are incorporated into the microfluidic configuration, while paper-based origami architectures are utilized for loading reagents and managing the flow of the filtered solution. Furthermore, lateral flow through capillary and passive micropump configurations is achieved using PDMS and adhesive tape microfluidics.

**Table 2:** Microfluidic electrochemical devices for biomarker detection in blood and serum

Target biomarker	Sensor	Microfluidic substrate	Microfluidic flow	Microfluidic device function	Detection technique	Linear range/LOD	Matrix	Ref
VEGF165	Magnetron sputtered Cr and Au on glass layer	PDMS on glass layer	Centrifugal forces	Plasma separation and directing to the sensing area	Differential pulse voltammetry	1.0–10^4^ pg/mL/0.67 pg/mL	Blood	90
Succinate	SPE on PET	Pressure-sensitive adhesive (PSA) layers for structuring fluidic channels and chambers (ARcare 8939)	Lateral flow	Plasma separation membrane housing and directing to the sensing area	Square wave voltammetry	50 nM–250 μM/5 nM	Blood	91
DNA of Human Papillomavirus	Commercial SPE (Metrohm)	PDMS	Capillary micropump and parallel capillaries	Plasma separation membrane housing and directing to the sensing area through tubes	Differential pulse voltammetry	1–10 μM/---	Blood	92
Ascorbic acid	Commercial SPE (PalmSens)	Whatman LF1 and Whatman grade 1 paper	Lateral flow	Plasma separation and directing to the sensing area	Cyclic voltammetry	0–80 μM/2.44 μM	Blood	93
Antibodies anti-SARS-CoV-2 nucleocapsid protein	Stencil-printed electrode on polyester	Double-sided adhesive, PET, nitrocellulose, Whatman grade 1 filter paper (waste pad)	Lateral flow passive pump by waste pad	Plasma separation membrane housing, immunoassay performing and directing to the sensing area	Chronoamperometry	---/5 ng/mL (in blood)	Blood	94
C-reactive protein and prealbumin	Screen-printed electrode on Whatman paper	Whatman grade 1 filter paper	Vertical flow origami system	Plasma separation membrane housing and vertical directing to the sensing area	Differential pulse voltammetry	C-reactive protein: 5–10^6^ pg/mL/5 pg/mL Prealbumin: 10–10^6^ pg/mL/10 pg/mL	Blood	95
Cardiac troponin I, brain natriuretic peptide-32, and d-dimer	SPE on cellulose Whatman grade 1 filter paper	Plasma separation membrane, Whatman CHR 3MM (absorption pad), and Whatman grade 1 filter paper (SPE)	Vertical flow on origami system	Plasma separation membrane housing, absorption paper holding and vertical directing to the sensing area	Electrochemical impedance spectroscopy	(In artificial blood plasma) Cardiac troponin I: 10 pg/mL-100 ng/mL /4.6 pg/mL Brain natriuretic peptide: 1 pg/mL to 1 ng/mL/1.2 pg/mL d-dimer: 1 ng/mL–10 μg/mL/146 pg/mL	Blood	96
Physostigmine, rivastigmine, and donepezil	SPE on office paper Fabriano Copy 2	Office paper Fabriano Copy 2, and Cordenons filter paper, plasma separation membrane	Vertical flow	Plasma separation, reagent loading, and vertical directing to the sensing area	Amperometry	Physostigmine: 0.01–0.5 µM/0.009 µM Rivastigmine: 0.5–25 µM/0.4 µM Donepezil: 0.5–30 µM/0.3 µM	Blood	97
Carcinoembryonic antigen	SPE on Whatman paper	Whatman grade 1 filter paper	Lateral and vertical flow	Molecular Imprinted Polymer synthesis and immunoassay performing	Chronoamperometry	1–500 ng/mL/0.32 ng/mL	Serum	100
α‑Fetoprotein	SPE on Whatman paper	Whatman grade 4 paper	Rotating flow by folded circle paper pads and vertical flow	Assay performing and directing to the sensing area	Electrochemical impedance spectroscopy	0.5 pg/mL–10 ng/mL/3.54 fg/mL	Serum	101
Hepatitis B virus DNA	SPE on PVC	Not specified waxed pad, sample pad and absorbent pad, nitrocellulose	Lateral flow	Assay performing and directing to the sensing area	Anodic stripping Voltammetry	10 pM–2 µM/7.23 pM	Serum	102
Bilirubin (conjugated and unconjugated ones)	LIG electrode on polyimide	Glass fiber from diagnostic pad	Lateral flow	Directing to two sensing areas, reagent loading	Square wave voltammetry	100–400 μM/---	Serum	103
Carcinoembryonic antigen and neuron-specific enolase	SPE on Whatman paper	Whatman grade 1 filter paper	Lateral flow on multilayered platform	Directing to two sensing areas	Differential pulse voltammetry	Carcinoembryonic antigen: 0.01–500 ng/mL/2 pg/mL Neuron-specific enolase: 0.05–500 ng/mL/10 pg/mL	Serum	104
HBsAg and HCVcAg	SPE on transparency film	Whatman no. 1 filter paper with transparency films and double-sided adhesive tape	Lateral flow	Directing to two sensing areas, reagent adding	Chronoamperometry	HBsAg: 0.1–250 ng/mL/18.2 pg/mL HCVcAg: 0.001–250 ng/mL/1.19 pg/mL	Serum	105
Transferrin	Stencil-printed electrodes on Whatman paper	Whatman chromatography paper 1 CHR	Lateral flow	Directing to two sensing areas	Square wave voltammetry	0.25–10 g/L/0.061 g/L	Serum	106
Dopamine and uric acid	LIG on polyimide	Polyimide	Flow by capillarity	Directing to the sensing areas	Differential pulse voltammetry	Dopamine: 0.91–171.15 μM/72 nM Uric acid: 0.83–250.04 μM for UA μM/48 nM	Serum	61
Glucose, uric acid, and sarcosine	Micropillar array electrodes	Double-sided adhesive tape	Manual syringe	Directing to the sensing areas	Amperometry	Glucose: 0.1–12 mM/58.5 µM Uric acid: 10–800 μM/3.4 µM Sarcosine: 2.5–100 μM/0.4 µM	Serum	107
α1-acid glycoprotein	Stencil-printed electrode on transparent film	Double-sided adhesive and transparent film (Samsill)	Capillary-driven flow and paper pump	Assay performing and directing to the sensing area	Square wave voltammetry	500–2000 mg/L/231 mg/L	Serum	108
Glucose	Commercial SPE (Yituo Biosensing Technology)	PDMS	Capillary micropumps	Housing of reagent-loaded paper	Amperometry	1 μM–1 mM and 1–6 mM/0.32 μM	Serum	109

*HBsAg* hepatitis B virus core antigen, *HCVcAg* hepatitis C virus core antigen, *LIG* laser-induced graphene, *PDMS* polydimethylsiloxane, *PET* polyethylene terephthalate, *PVC* poly(vinyl chloride), *SPE* screen-printed electrode, *VEGF165* vascular endothelial growth factor 165

The seamless composition of serum, owing to the lower presence/absence of lipidic and protein substances, enables the use of microfluidic channels for various purposes. This includes focusing on performing the assay and directing the sample to dual sensing zones for multiparametric detection (Table 3). This is mainly why paper is often chosen as the substrate to create electrochemical microfluidic platforms applied for analyte detection in serum; no highly resistant structures are needed, nor any pretreatment of the sample.

**Table 3:** Microfluidic electrochemical devices for biomarker detection in saliva and urine

Target biomarker	Sensor	Microfluidic substrate	Microfluidic flow	Microfluidic device function	Detection technique	Linear range/LOD	Matrix	Ref
Interleukin-8, tumor necrosis factor-α, and myeloperoxidase	Transducer array chip	Whatman 1 chromatographic paper	Lateral flow	Addiction of sample matrix, magneto-immunoassay performing	Amperometry	Interleukin-8: 0–2000 pg/mL/420 pg/mL Necrosis factor-α: 0–5000 pg/mL/174 pg/mL Myeloperoxidase: 0–5000 ng/mL/116 ng/mL	Saliva	110
Uric acid	SPE	PET	Manual syringe	Enabling filtering at the inlet channel, delivering to sensing area	Cyclic voltammetry	5–500 μM/4.2 μM	Saliva	111
Glucose, lactate, cholesterol, and uric acid	SPE on PET	PDMS	Flow by horizontal capillary channels	Enabling filtering at the inlet channel, delivering to sensing area	Amperometry	Glucose: 100–1400 µM/20.6 µM Lactate: 50–1600 µM/15 µM Cholesterol: 50–900 µM/30.5 µM Uric acid: 0.2–1.6 mM/62.5 µM	Saliva	112
Thiocyanate	SPE on paper	Whatman no. 1 filter paper	Flow by hollow capillary channel as a micropump	Delivering to sensing areas	Square wave voltammetry	0.5–100 mM/0.2 mM	Saliva	113
Uric acid and Ca^2+^	LIG electrode on polyimide	Polyimide	Flow by capillarity	Delivering to sensing areas	Uric acid: differential pulse voltammetry Ca^2+^: potentiometry	Uric acid: 10–50 μM/217 nM Ca^2+^: 10^−5^–10^−2.5^ M/10^−5.3^ M	Saliva	60
Human chorionic gonadotropin	SPE on PET	Plastic cartridge	Gravity force, waste pad	Directing to sensing area, immunoassay step performing	Amperometry	0–25 mIU/mL/2.17 mIU/mL	Urine	114
Glucose	SPE on polyester	Whatman #1 paper	Lateral flow	Directing to multiple sensing areas	Amperometry	0.1–40 mM/0.03 mM	Urine	116
Creatinine, uric acid, and glucose	SPE on polyester	Whatman #1 paper	Lateral flow	Directing to multiple sensing areas	Creatinine and uric acid: differential pulse voltammetry Glucose: amperometry	Creatinine: 0.106 to 4.5 mM/0.084 mM Uric acid: 0.05–3 mM/0.012 mM Glucose: 0.625–20 mM/0.12 mM	Urine	115
Pb^2+^	Gold-plated plastic electrode	Whatman not specified	Lateral flow	Loading of reagent and filtering	Square wave-anodic stripping voltammetry	10–500 ppb/9 ppb (artificial urine)	Urine	117

*LIG* laser-induced graphene, *PDMS* polydimethylsiloxane, *PET* polyethylene terephthalate, *SPE* screen-printed electrode

Similarly, the application for analyte detection in saliva and urine mainly focuses on directing the fluid to the sensing area (Table 3). This is accomplished mainly by exploiting capillary channels in plastic supports and cellulose network capillarity in paper-based microfluidics.

Analytical features of the discussed microfluidic sensing devices reported in the tables demonstrate the reliability of these sensing platforms when used for quantitative analyses.

Despite these recent applications, several challenges still persist. Among the crucial issues, the standardization of fabrication methods is the main drawback. Currently, the manufacturing process is still restricted to research applications, limiting scalability and increasing production costs, hampering widespread commercial use. Additionally, the typical fabrication procedures usually deliver disposable devices, with consequences on the reproducibility of microfluidic performance and long-term stability of integrated electrodes.

Alongside this, each substrate material is characterized by benefits and downsides.

Paper, for instance, offers undeniable advantages related to cost-effective and sustainable production, alongside smooth fluid motion due to capillary action. However, it is fragile and prone to tearing or deformation, hindering long-term application. Additionally, while creating channels using wax-printed hydrophobic barriers is a straightforward and fast process, it results in low microchannel definition, making it challenging to build complex networks.

PDMS substrate is characterized by flexibility and robustness, but fabrication requires fluid dynamics studies to design the microchannels, the fluid motion being governed by valve and capillary micropumps. Additionally, the whole fabrication process is more labor-intensive and costly, making mass production difficult.

Lastly, adhesive tape is a cost-effective solution that allows for easy and quick assembly of devices through layer stacking. Nonetheless, it suffers from adhesive degradation over time, which can lead to leaching.

Looking ahead in the future, further developments are still needed, aimed at enhancing the integration with last-generation technologies, including artificial intelligence, wireless data transmission, and high-throughput readout systems.

Indeed, leveraging machine learning can boost the interpretation of data, which is beneficial for both optimizing microfluidic fabrication processes and improving the reliability of sensor devices, especially in complex clinical scenarios.

The integration with digital health technologies will advance analytical tools towards IoT data, facilitating real-time communication between patients and medical professionals.

Additionally, employing 3D-printing to create supporting structures can promote the integration of microfluidics and electrochemical sensors, thereby increasing the robustness of the platform and streamlining the entire fabrication process.

In conclusion, microfluidic electrochemical sensing tools are rapidly evolving into versatile and reliable instruments for next-generation POC analytical platforms. Further advancements, boosted by cross-disciplinary innovation and high-throughput technologies, are ready to revolutionize the execution and location of medical diagnostics, making healthcare more accessible to patients than ever before.

## Data Availability

No data were created or analyzed in this study.

## References

[1] Nicolás D, Coloma E, Pericàs JM. Alternatives to conventional hospitalisation that enhance health systems’ capacity to treat COVID-19. Lancet Infect Dis. 2021;21:591–3. 10.1016/S1473-3099(21)00093-1. 33711274 10.1016/S1473-3099(21)00093-1 PMC8063075

[2] Pericàs JM, Cucchiari D, Torrallardona-Murphy O, Calvo J, Serralabós J, Alvés E, et al. Hospital at home for the management of COVID-19: preliminary experience with 63 patients. Infection. 2021;49:327–32. 10.1007/s15010-020-01527-z. 32995970 10.1007/s15010-020-01527-z PMC7523688

[3] Gonçalves-Bradley DC, Iliffe S, Doll HA, Broad J, Gladman J, Langhorne P, Richards SH, Shepperd S. Early discharge hospital at home. Cochrane Database of Systematic Reviews. 201710.1002/14651858.CD000356.pub4 10.1002/14651858.CD000356.pub4 PMC6481686 28651296

[4] Land KJ, Boeras DI, Chen X-S, Ramsay AR, Peeling RW. Reassured diagnostics to inform disease control strategies, strengthen health systems and improve patient outcomes. Nat Microbiol. 2018;4:46–54. 10.1038/s41564-018-0295-3. 30546093 10.1038/s41564-018-0295-3 PMC7097043

[5] Doganay MT, Chakraborty P, Bommakanti SM, Jammalamadaka S, Battalapalli D, Madabhushi A, et al. Artificial intelligence performance in testing microfluidics for point-of-care. Lab Chip. 2024;24:4998–5008. 10.1039/D4LC00671B. 39360887 10.1039/d4lc00671b PMC11448392

[6] Han G-R, Goncharov A, Eryilmaz M, Ye S, Palanisamy B, Ghosh R, et al. Machine learning in point-of-care testing: innovations, challenges, and opportunities. Nat Commun. 2025;16:3165. 10.1038/s41467-025-58527-6. 40175414 10.1038/s41467-025-58527-6 PMC11965387

[7] Xu J, Tao X, Liu X, Yang L. Wearable eye patch biosensor for noninvasive and simultaneous detection of multiple biomarkers in human tears. Anal Chem. 2022;94:8659–67. 10.1021/acs.analchem.2c00614. 35656772 10.1021/acs.analchem.2c00614

[8] Pan J, Zhang R, Xia S, Yang M, Jiang L, Yi C. High-throughput point-of-care testing using the smartphone-based, cloud computing-enabled ECL analyzer and single ITO electrode-based sensing chips. Microchem J. 2025;209:112707. 10.1016/j.microc.2025.112707.

[9] Behera PP, Kumar N, Kumari M, Kumar S, Mondal PK, Arun RK. Integrated microfluidic devices for point-of-care detection of bio-analytes and disease. Sens Diagn. 2023;2:1437–59. 10.1039/D3SD00170A.

[10] Mitchell KR, Esene JE, Woolley AT. Advances in multiplex electrical and optical detection of biomarkers using microfluidic devices. Anal Bioanal Chem. 2022;414:167–80. 10.1007/s00216-021-03553-8. 34345949 10.1007/s00216-021-03553-8 PMC8331214

[11] Microfluidics market size booms at 12.50% CAGR by 2034. https://www.towardshealthcare.com/insights/microfluidics-market-sizing#:~:text=in%20the%20Report-,Microfluidics%20Market%20Size%2C%20Technologies%2C%20Drivers%20and%20Recent%20Announcements,period%20from%202025%20to%202034. Accessed 16 Jun 2025

[12] Rios A, Escarpa A, Simonet B. Miniaturization of analytical systems: principles, designs and applications. 1st ed. Wiley; 2009.

[13] Rackus DG, Shamsi MH, Wheeler AR. Electrochemistry, biosensors and microfluidics: a convergence of fields. Chem Soc Rev. 2015;44:5320–40. 10.1039/C4CS00369A. 25962356 10.1039/c4cs00369a

[14] Mohan JM, Amreen K, Javed A, Dubey SK, Goel S. Emerging trends in miniaturized and microfluidic electrochemical sensing platforms. Curr Opin Electrochem. 2022;33:100930. 10.1016/j.coelec.2021.100930.

[15] Dai Z. Recent advances in the development of portable electrochemical sensors for controlled substances. Sensors (Basel). 2023;23:3140. 10.3390/s23063140. 36991851 10.3390/s23063140 PMC10058808

[16] Colozza N, Chebil A, Arduini F. Smartphone-integrated electrochemical (bio)sensors as smart and reliable analytical tools. In: Comprehensive analytical chemistry. Elsevier, 2023. pp 73–108. 10.1016/bs.coac.2022.12.002

[17] Whitesides GM. The origins and the future of microfluidics. Nature. 2006;442:368–73. 10.1038/nature05058. 16871203 10.1038/nature05058

[18] Smith S, Sypabekova M, Kim S. Double-sided tape in microfluidics: a cost-effective method in device fabrication. Biosensors. 2024;14:249. 10.3390/bios14050249. 38785723 10.3390/bios14050249 PMC11118809

[19] Bezinge L, Shih C, Richards DA, deMello AJ. Electrochemical paper‐based microfluidics: harnessing capillary flow for advanced diagnostics. Small 2024:2401148. 10.1002/smll.202401148. 10.1002/smll.202401148 38801400

[20] Holman JB, Shi Z, Fadahunsi AA, Li C, Ding W. Advances on microfluidic paper-based electroanalytical devices. Biotechnol Adv. 2023;63:108093. 10.1016/j.biotechadv.2022.108093. 36603801 10.1016/j.biotechadv.2022.108093

[21] Shen L, Zhang G, Etzold BJM. Paper-based microfluidics for electrochemical applications. ChemElectroChem. 2020;7:10–30. 10.1002/celc.201901495. 32025468 10.1002/celc.201901495 PMC6988477

[22] Alghannam F, Alayed M, Alfihed S, Sakr MA, Almutairi D, Alshamrani N, et al. Recent progress in PDMS-based microfluidics toward integrated organ-on-a-chip biosensors and personalized medicine. Biosensors. 2025;15:76. 10.3390/bios15020076. 39996978 10.3390/bios15020076 PMC11852457

[23] Tavakoli H, Mohammadi S, Li X, Fu G, Li X. Microfluidic platforms integrated with nano-sensors for point-of-care bioanalysis. TrAC Trends Anal Chem. 2022;157:116806. 10.1016/j.trac.2022.116806. 10.1016/j.trac.2022.116806 PMC10621318 37929277

[24] Fattahi Z, Hasanzadeh M. Nanotechnology-assisted microfluidic systems for chemical sensing, biosensing, and bioanalysis. TrAC Trends Anal Chem. 2022;152:116637. 10.1016/j.trac.2022.116637.

[25] Ahmad MM, Ma Y, Badshah M, Ali S, Idrees M, Ismail MA, et al. 2D-MXenes: progress in synthesis, intercalation, and applications in microfluidic sensors. Surf Interface. 2025;56:105678. 10.1016/j.surfin.2024.105678.

[26] Shukhratovich Abdullaev S, H Althomali R, Raza Khan A, Sanaan Jabbar H, Abosoda M, Ihsan A, et al. Integrating of analytical techniques with enzyme-mimicking nanomaterials for the fabrication of microfluidic systems for biomedical analysis. Talanta 2024;273:125896. 10.1016/j.talanta.2024.125896. 10.1016/j.talanta.2024.125896 38479027

[27] Yamaguchi H, Miyazaki M. Enzyme-immobilized microfluidic devices for biomolecule detection. TrAC Trends Anal Chem. 2023;159:116908. 10.1016/j.trac.2022.116908.

[28] Akyazi T, Basabe-Desmonts L, Benito-Lopez F. Review on microfluidic paper-based analytical devices towards commercialisation. Anal Chim Acta. 2018;1001:1–17. 10.1016/j.aca.2017.11.010. 29291790 10.1016/j.aca.2017.11.010

[29] Kumari M, Gupta V, Kumar N, Arun RK. Microfluidics-based nanobiosensors for healthcare monitoring. Mol Biotechnol. 2024;66:378–401. 10.1007/s12033-023-00760-9. 37166577 10.1007/s12033-023-00760-9 PMC10173227

[30] Li Z, Liu H, Wang D, Zhang M, Yang Y, Ren T. Recent advances in microfluidic sensors for nutrients detection in water. TrAC Trends Anal Chem. 2023;158:116790. 10.1016/j.trac.2022.116790.

[31] Sekhwama M, Mpofu K, Sivarasu S, Mthunzi-Kufa P. Applications of microfluidics in biosensing. Discov Appl Sci. 2024;6:303. 10.1007/s42452-024-05981-4.

[32] Madadelahi M, Romero-Soto FO, Kumar R, Tlaxcala UB, Madou MJ. Electrochemical sensors: types, applications, and the novel impacts of vibration and fluid flow for microfluidic integration. Biosens Bioelectron. 2025;272:117099. 10.1016/j.bios.2024.117099. 39764983 10.1016/j.bios.2024.117099

[33] Garg M, Pamme N. Microfluidic (bio)-sensors based on 2-D layered materials. TrAC Trends Anal Chem. 2023;158:116839. 10.1016/j.trac.2022.116839.

[34] Zhang Y, Li J, Jiao S, Li Y, Zhou Y, Zhang X, et al. Microfluidic sensors for the detection of emerging contaminants in water: a review. Sci Total Environ. 2024;929:172734. 10.1016/j.scitotenv.2024.172734. 38663621 10.1016/j.scitotenv.2024.172734

[35] Khalaf EM, Sanaan Jabbar H, Mireya Romero-Parra R, Raheem Lateef Al-Awsi G, Setia Budi H, Altamimi AS, et al. Smartphone-assisted microfluidic sensor as an intelligent device for on-site determination of food contaminants: developments and applications. Microchem. J. 2023;190:108692. 10.1016/j.microc.2023.108692.

[36] Park S, Kim S, Lee S, Tsukruk VV, Park S, Lim H. Advanced microfluidic-based wearable electrochemical sensors for continuous biochemical monitoring. Adv Electron Mater. 2025;11:2500010. 10.1002/aelm.202500010.

[37] Liu C, Sun X, Wang Q, Wang S, Wang Q, Zhang S. State of the art overview wearable microfluidic noninvasive biomarker sensors for sweat analysis. Microchem J. 2025;209:112847. 10.1016/j.microc.2025.112847.

[38] Sanati A, Esmaeili Y, Bidram E, Shariati L, Rafienia M, Mahshid S, et al. Recent advancement in electrode materials and fabrication, microfluidic designs, and self-powered systems for wearable non-invasive electrochemical glucose monitoring. Appl Mater Today. 2022;26:101350. 10.1016/j.apmt.2021.101350.

[39] Hayatu S, Audu AA, Ladan M. Recent development in drugs of abuse detection: from electrochemical sensors to microfluidic coupled electrochemical sensors. Chem Afr. 2024;7:1783–801. 10.1007/s42250-024-00885-7.

[40] Asci Erkocyigit B, Ozufuklar O, Yardim A, Guler Celik E, Timur S. Biomarker detection in early diagnosis of cancer: recent achievements in point-of-care devices based on paper microfluidics. Biosensors. 2023;13:387. 10.3390/bios13030387. 36979600 10.3390/bios13030387 PMC10046104

[41] Liu Z, Zhou Y, Lu J, Gong T, Ibáñez E, Cifuentes A, et al. Microfluidic biosensors for biomarker detection in body fluids: a key approach for early cancer diagnosis. Biomark Res. 2024;12:153. 10.1186/s40364-024-00697-4. 39639411 10.1186/s40364-024-00697-4 PMC11622463

[42] Didarian R, Azar MT. Microfluidic biosensors: revolutionizing detection in DNA analysis, cellular analysis, and pathogen detection. Biomed Microdevices. 2025;27:10. 10.1007/s10544-025-00741-6. 40011268 10.1007/s10544-025-00741-6

[43] Pakeeza, Draz MU, Yaqub A, Jafry AT, Khan M, Ajab H. Electrochemical sensing of B-complex vitamins: current challenges and future prospects with microfluidic integration. RSC Adv 2024;14:10331–47. 10.1039/D4RA00555D. 10.1039/d4ra00555d PMC10977047 38549795

[44] Zarean Mousaabadi K, Talebi Vandishi Z, Kermani M, Arab N, Ensafi AA. Recent developments toward microfluidic point-of-care diagnostic sensors for viral infections. TrAC Trends Anal Chem. 2023;169:117361. 10.1016/j.trac.2023.117361.

[45] Dungchai W, Chailapakul O, Henry CS. Electrochemical detection for paper-based microfluidics. Anal Chem. 2009;81:5821–6. 10.1021/ac9007573. 19485415 10.1021/ac9007573

[46] Martinez AW, Phillips ST, Whitesides GM, Carrilho E. Diagnostics for the developing world: microfluidic paper-based analytical devices. Anal Chem. 2010;82:3–10. 10.1021/ac9013989. 20000334 10.1021/ac9013989

[47] Colozza N, Caratelli V, Moscone D, Arduini F. Origami paper-based electrochemical (bio)sensors: state of the art and perspective. Biosensors. 2021;11:328. 10.3390/bios11090328. 34562920 10.3390/bios11090328 PMC8467589

[48] Böhm A, Carstens F, Trieb C, Schabel S, Biesalski M. Engineering microfluidic papers: effect of fiber source and paper sheet properties on capillary-driven fluid flow. Microfluid Nanofluid. 2014;16:789–99. 10.1007/s10404-013-1324-4.

[49] Colozza N, Mazzaracchio V, Arduini F. Paper-based electrochemical (bio)sensors for the detection of target analytes in liquid, aerosol, and solid samples. Annu Rev Anal Chem. 2024;17:127–47. 10.1146/annurev-anchem-061522-034228. 10.1146/annurev-anchem-061522-034228 38640070

[50] Adkins J, Boehle K, Henry C. Electrochemical paper-based microfluidic devices. Electrophoresis. 2015;36:1811–24. 10.1002/elps.201500084. 25820492 10.1002/elps.201500084

[51] Miranda I, Souza A, Sousa P, Ribeiro J, Castanheira EMS, Lima R, et al. Properties and applications of PDMS for biomedical engineering: a review. JFB. 2021;13:2. 10.3390/jfb13010002. 35076525 10.3390/jfb13010002 PMC8788510

[52] Love JC, Anderson JR, Whitesides GM. Fabrication of three-dimensional microfluidic systems by soft lithography. MRS Bull. 2001;26:523–8. 10.1557/mrs2001.124.

[53] Goral VN, Hsieh Y-C, Petzold ON, Faris RA, Yuen PK. Hot embossing of plastic microfluidic devices using poly(dimethylsiloxane) molds. J Micromech Microeng. 2011;21(1):017002. 10.1088/0960-1317/21/1/017002.

[54] Lee UN, Su X, Guckenberger DJ, Dostie AM, Zhang T, Berthier E, et al. Fundamentals of rapid injection molding for microfluidic cell-based assays. Lab Chip. 2018;18:496–504. 10.1039/C7LC01052D. 29309079 10.1039/c7lc01052d PMC5790604

[55] CC Toepke MW, Beebe DJ. PDMS absorption of small molecules and consequences in microfluidic applications. Lab Chip 2006;6:1484. 10.1039/b612140c. 10.1039/b612140c 17203151

[56] Wang JD, Douville NJ, Takayama S, ElSayed M. Quantitative analysis of molecular absorption into PDMS microfluidic channels. Ann Biomed Eng. 2012;40:1862–73. 10.1007/s10439-012-0562-z. 22484830 10.1007/s10439-012-0562-z

[57] Raj MK, Chakraborty S. PDMS microfluidics: a mini review. J Appl Polym Sci. 2020;137:48958. 10.1002/app.48958.

[58] Liao C, Li S, Yang C, Du C, Yao H, Han Z, et al. Wearable epidermal sensor patch with biomimetic microfluidic channels for fast and time-sequence monitoring of sweat glucose and lactate. Talanta. 2025;287:127683. 10.1016/j.talanta.2025.127683. 39923668 10.1016/j.talanta.2025.127683

[59] Zhong B, Qin X, Xu H, Liu L, Li L, Li Z, et al. Interindividual- and blood-correlated sweat phenylalanine multimodal analytical biochips for tracking exercise metabolism. Nat Commun. 2024;15:624. 10.1038/s41467-024-44751-z. 38245507 10.1038/s41467-024-44751-z PMC10799919

[60] Johnson ZT, Ellis G, Pola CC, Banwart C, McCormick A, Miliao GL, Duong D, Opare‐Addo J, Sista H, Smith EA, Hu H, Gomes CL, Claussen JC. Enhanced laser‐induced graphene microfluidic integrated sensors (LIGMIS) for on‐site biomedical and environmental monitoring. Small. 2025;2500262. 10.1002/smll.202500262 10.1002/smll.202500262 PMC12366263 40195914

[61] Chen X, Peng W, Yao L, Lian H, Liu B, Wei X. Modification-free pumpless laser-induced graphene multichannel open microfluidic electrode chip for simultaneous quantification of dual targets. Sens Actuat B-Chem. 2024;400:134905. 10.1016/j.snb.2023.134905.

[62] Vinoth R, Nakagawa T, Mathiyarasu J, Mohan AMV. Fully printed wearable microfluidic devices for high-throughput sweat sampling and multiplexed electrochemical analysis. ACS Sens. 2021;6:1174–86. 10.1021/acssensors.0c02446. 33517662 10.1021/acssensors.0c02446

[63] Hu M, Wang Z, Zhang L, Lin S, Liao J. A microfluidic patch for wireless wearable electrochemical detection of sweat metabolites. Sens Actuat B-Chem. 2025;422:136604. 10.1016/j.snb.2024.136604.

[64] Xuan X, Pérez-Ràfols C, Chen C, Cuartero M, Crespo GA. Lactate biosensing for reliable on-body sweat analysis. ACS Sens. 2021;6:2763–71. 10.1021/acssensors.1c01009. 34228919 10.1021/acssensors.1c01009 PMC8397467

[65] Ying Z, Qiao L, Liu B, Gao L, Zhang P. Development of a microfluidic wearable electrochemical sensor for the non-invasive monitoring of oxidative stress biomarkers in human sweat. Biosens Bioelectron. 2024;261:116502. 10.1016/j.bios.2024.116502. 38896980 10.1016/j.bios.2024.116502

[66] Fiore L, Mazzaracchio V, Serani A, Fabiani G, Fabiani L, Volpe G, et al. Microfluidic paper-based wearable electrochemical biosensor for reliable cortisol detection in sweat. Sensors and Actuators B: Chemical. 2023;379:133258. 10.1016/j.snb.2022.133258.

[67] Fiore L, Mazzaracchio V, Antinucci A, Ferrara R, Sciarra T, Lista F, et al. Wearable electrochemical device based on butterfly-like paper-based microfluidics for pH and Na+ monitoring in sweat. Microchim Acta. 2024;191:580. 10.1007/s00604-024-06564-1. 10.1007/s00604-024-06564-1 PMC11380643 39243287

[68] Saha T, Songkakul T, Knisely CT, Yokus MA, Daniele MA, Dickey MD, et al. Wireless wearable electrochemical sensing platform with zero-power osmotic sweat extraction for continuous lactate monitoring. ACS Sens. 2022;7:2037–48. 10.1021/acssensors.2c00830. 35820167 10.1021/acssensors.2c00830

[69] Cao Q, Liang B, Tu T, Wei J, Fang L, Ye X. Three-dimensional paper-based microfluidic electrochemical integrated devices (3D-PMED) for wearable electrochemical glucose detection. RSC Adv. 2019;9:5674–81. 10.1039/C8RA09157A. 35515907 10.1039/c8ra09157a PMC9060762

[70] Li M, Wang L, Liu R, Li J, Zhang Q, Shi G, et al. A highly integrated sensing paper for wearable electrochemical sweat analysis. Biosens Bioelectron. 2021;174:112828. 10.1016/j.bios.2020.112828. 33250335 10.1016/j.bios.2020.112828

[71] Zhang S, Wang H, Zheng Y, Yao Y, Li T, Ma Y, Zhou Y, Chen Z, Wei Y, Fang L, Chen X, Ye X, Zhou J, Liang B. An integrated paper‐based patch for wearable detection of diabetic nephropathy biomarkers in sweat. Adv Funct Materials. 2025;2501970. 10.1002/adfm.202501970

[72] Cao Q, Liang B, Mao X, Wei J, Tu T, Fang L, et al. A smartwatch integrated with a paper-based microfluidic patch for sweat electrolytes monitoring. Electroanalysis. 2021;33:643–51. 10.1002/elan.202060025.

[73] Zhang Y, Jin C, Wang C, Zeng X, Yang M, Hou C, et al. Fe/Pt dual-atom catalyst-enabled wearable microfluidic patch for superior uric acid detection in sweat. Biosens Bioelectron. 2025;271:117001. 10.1016/j.bios.2024.117001. 39673956 10.1016/j.bios.2024.117001

[74] Peng H-L, Zhang Y, Liu H, Gao C. Flexible wearable electrochemical sensors based on AuNR/PEDOT:PSS for simultaneous monitoring of levodopa and uric acid in sweat. ACS Sens. 2024;9:3296–306. 10.1021/acssensors.4c00649. 38829039 10.1021/acssensors.4c00649

[75] Yin Y, Tan Z, Zhu W, Pu Z, Yu H, Wang R, et al. A wearable microfluidic system for efficient sweat collection and real-time detection. Talanta. 2024;274:125967. 10.1016/j.talanta.2024.125967. 38537349 10.1016/j.talanta.2024.125967

[76] Zhang S, Zahed MA, Sharifuzzaman Md, Yoon S, Hui X, Chandra Barman S, et al. A wearable battery-free wireless and skin-interfaced microfluidics integrated electrochemical sensing patch for on-site biomarkers monitoring in human perspiration. Biosens Bioelectron. 2021;175:112844. 10.1016/j.bios.2020.112844. 33248878 10.1016/j.bios.2020.112844

[77] Sun T, Hui J, Zhou L, Lin B, Sun H, Bai Y, et al. A low-cost and simple-fabricated epidermal sweat patch based on “cut-and-paste” manufacture. Sensors and Actuators B: Chemical. 2022;368:132184. 10.1016/j.snb.2022.132184.

[78] Niu J, Lin S, Chen D, Wang Z, Cao C, Gao A, et al. A fully elastic wearable electrochemical sweat detection system of tree-bionic microfluidic structure for real-time monitoring. Small. 2024;20:2306769. 10.1002/smll.202306769. 10.1002/smll.202306769 37932007

[79] Xu W, Cao Y, Shi H, Jia X, Zheng Y, Tan Z, et al. Skin-interfaced sweat monitoring patch constructed by flexible microfluidic capillary pump and Cu-MOF sensitized electrochemical sensor. Talanta. 2025;291:127895. 10.1016/j.talanta.2025.127895. 40056654 10.1016/j.talanta.2025.127895

[80] Zhao H, Zhang X, Qin Y, Xia Y, Xu X, Sun X, et al. An integrated wearable sweat sensing patch for passive continuous analysis of stress biomarkers at rest. Adv Funct Mater. 2023;33:2212083. 10.1002/adfm.202212083.

[81] Mei X, Chen Z, Wen A, Zhang J, Wei X, Wang F, et al. Wearable three-dimensional paper-based microfluidic electrochemical sensors for real-time sweat monitoring. Chem Eng J. 2025;515:163786. 10.1016/j.cej.2025.163786.

[82] Bandodkar AJ, Gutruf P, Choi J, Lee K, Sekine Y, Reeder JT, et al. Battery-free, skin-interfaced microfluidic/electronic systems for simultaneous electrochemical, colorimetric, and volumetric analysis of sweat. Sci Adv. 2019;5:eaav3294. 10.1126/sciadv.aav3294. 30746477 10.1126/sciadv.aav3294 PMC6357758

[83] Fu J, Wang Y, Ding Y, Wang J, Deng S, Jiang Z, et al. Wearable ring sensor for monitoring biomarkers of atherosclerosis in sweat. Talanta. 2025;287:127608. 10.1016/j.talanta.2025.127608. 39827480 10.1016/j.talanta.2025.127608

[84] Sun T, Hui J, Lin B, Sun H, Zhou L, Zhao J, et al. Sequential biofluid sampling microfluidic multi-sensing patch for more accurate sweat analysis under sedentary condition. Appl Mater Today. 2023;34:101910. 10.1016/j.apmt.2023.101910.

[85] Bolat G, De La Paz E, Azeredo NF, Kartolo M, Kim J, De Loyola E Silva AN, Rueda R, Brown C, Angnes L, Wang J, Sempionatto JR. Wearable soft electrochemical microfluidic device integrated with iontophoresis for sweat biosensing. Anal Bioanal Chem. 2022;414:5411–5421. 10.1007/s00216-021-03865-9 10.1007/s00216-021-03865-9 35015101

[86] Peringeth K, Ganguly A, Pal A, Roy Chowdhury J, Kaswan K, Ho H-Y, et al. Self-powered microfluidic-based sensor for noninvasive sweat analysis. Sensors and Actuators B: Chemical. 2025;423:136859. 10.1016/j.snb.2024.136859.

[87] Lin H, Zhao Y, Lin S, Wang B, Yeung C, Cheng X, et al. A rapid and low-cost fabrication and integration scheme to render 3D microfluidic architectures for wearable biofluid sampling, manipulation, and sensing. Lab Chip. 2019;19:2844–53. 10.1039/C9LC00418A. 31359008 10.1039/c9lc00418a

[88] Lin H, Tan J, Zhu J, Lin S, Zhao Y, Yu W, et al. A programmable epidermal microfluidic valving system for wearable biofluid management and contextual biomarker analysis. Nat Commun. 2020;11:4405. 10.1038/s41467-020-18238-6. 32879320 10.1038/s41467-020-18238-6 PMC7467936

[89] Zhao Y, Wang B, Hojaiji H, Wang Z, Lin S, Yeung C, et al. A wearable freestanding electrochemical sensing system. Sci Adv. 2020;6:eaaz0007. 10.1126/sciadv.aaz0007. 32219164 10.1126/sciadv.aaz0007 PMC7083607

[90] He X, Wang X, Ge C, Li S, Wang L, Xu Y. Detection of VEGF165 in whole blood by differential pulse voltammetry based on a centrifugal microfluidic chip. ACS Sens. 2022;7:1019–26. 10.1021/acssensors.1c02641. 35362948 10.1021/acssensors.1c02641

[91] Farrokhnia M, Babamiri B, Mohammadi M, Sanati Nezhad A. MIP-chip: integrated microfluidic plasma separation and redox-enhanced molecularly imprinted polymer succinate sensor for whole blood metabolite analysis. ACS Sens. 2025;10:3112–22. 10.1021/acssensors.5c00355. 40146186 10.1021/acssensors.5c00355

[92] Keyvani F, Debnath N, Ayman Saleh M, Poudineh M. An integrated microfluidic electrochemical assay for cervical cancer detection at point-of-care testing. Nanoscale. 2022;14:6761–70. 10.1039/D1NR08252C. 35506790 10.1039/d1nr08252c

[93] Gautam N, Verma R, Ram R, Singh J, Sarkar A. Development of a biodegradable microfluidic paper-based device for blood-plasma separation integrated with non-enzymatic electrochemical detection of ascorbic acid. Talanta. 2024;266:125019. 10.1016/j.talanta.2023.125019. 37544255 10.1016/j.talanta.2023.125019

[94] Samper IC, Sánchez-Cano A, Khamcharoen W, Jang I, Siangproh W, Baldrich E, et al. Electrochemical capillary-flow immunoassay for detecting anti-SARS-CoV-2 nucleocapsid protein antibodies at the point of care. ACS Sens. 2021;6:4067–75. 10.1021/acssensors.1c01527. 34694794 10.1021/acssensors.1c01527 PMC8565458

[95] Sun S, Luo J, Zhu Y, Kong F, Mao G, Ming T, et al. Multifunctional self-driven origami paper-based integrated microfluidic chip to detect CRP and PAB in whole blood. Biosens Bioelectron. 2022;208:114225. 10.1016/j.bios.2022.114225. 35358776 10.1016/j.bios.2022.114225

[96] Fu H, Qin Z, Li X, Pan Y, Xu H, Pan P, et al. Paper-based all-in-one origami nanobiosensor for point-of-care detection of cardiac protein markers in whole blood. ACS Sens. 2023;8:3574–84. 10.1021/acssensors.3c01221. 37705448 10.1021/acssensors.3c01221

[97] Caratelli V, Ciampaglia A, Guiducci J, Sancesario G, Moscone D, Arduini F. Precision medicine in Alzheimer’s disease: an origami paper-based electrochemical device for cholinesterase inhibitors. Biosens Bioelectron. 2020;165:112411. 10.1016/j.bios.2020.112411. 32729530 10.1016/j.bios.2020.112411

[98] Chinnamani MV, Hanif A, Kannan PK, Kaushal S, Sultan MJ, Lee N-E. Soft microfiber-based hollow microneedle array for stretchable microfluidic biosensing patch with negative pressure-driven sampling. Biosens Bioelectron. 2023;237:115468. 10.1016/j.bios.2023.115468. 37343311 10.1016/j.bios.2023.115468

[99] Prat-Trunas J, Arias-Alpizar K, Álvarez-Carulla A, Orio-Tejada J, Molina I, Sánchez-Montalvá A, et al. Paper-based microfluidic electro-analytical device (PMED) for magneto-assay automation: towards generic point-of-care diagnostic devices. Biosens Bioelectron. 2024;246:115875. 10.1016/j.bios.2023.115875. 38039728 10.1016/j.bios.2023.115875

[100] Qi J, Li B, Zhou N, Wang X, Deng D, Luo L, et al. The strategy of antibody-free biomarker analysis by in-situ synthesized molecularly imprinted polymers on movable valve paper-based device. Biosens Bioelectron. 2019;142:111533. 10.1016/j.bios.2019.111533. 31377573 10.1016/j.bios.2019.111533

[101] Yakoh A, Mehmeti E, Kalcher K, Chaiyo S. Hand-operated, paper-based rotational vertical-flow immunosensor for the impedimetric detection of α-fetoprotein. Anal Chem. 2022;94:5893–900. 10.1021/acs.analchem.2c00079. 35394293 10.1021/acs.analchem.2c00079

[102] Srisomwat C, Yakoh A, Chuaypen N, Tangkijvanich P, Vilaivan T, Chailapakul O. Amplification-free DNA sensor for the one-step detection of the hepatitis B virus using an automated paper-based lateral flow electrochemical device. Anal Chem. 2021;93:2879–87. 10.1021/acs.analchem.0c04283. 33326737 10.1021/acs.analchem.0c04283

[103] Ong V, Mohamed MA, Ma H, Al-Shami A, Khazaee Nejad S, Amirghasemi F, et al. Bilisense: an affordable sensor for on-site diagnosis of jaundice and prevention of kernicterus. Biosens Bioelectron. 2025;280:117386. 10.1016/j.bios.2025.117386. 40209646 10.1016/j.bios.2025.117386 PMC12721466

[104] Wang Y, Luo J, Liu J, Sun S, Xiong Y, Ma Y, et al. Label-free microfluidic paper-based electrochemical aptasensor for ultrasensitive and simultaneous multiplexed detection of cancer biomarkers. Biosens Bioelectron. 2019;136:84–90. 10.1016/j.bios.2019.04.032. 31039491 10.1016/j.bios.2019.04.032

[105] Boonkaew S, Yakoh A, Chuaypen N, Tangkijvanich P, Rengpipat S, Siangproh W, et al. An automated fast-flow/delayed paper-based platform for the simultaneous electrochemical detection of hepatitis B virus and hepatitis C virus core antigen. Biosens Bioelectron. 2021;193:113543. 10.1016/j.bios.2021.113543. 34416431 10.1016/j.bios.2021.113543

[106] Dortez S, Pacheco M, Gasull T, Crevillen AG, Escarpa A. A dual colorimetric-electrochemical microfluidic paper-based analytical device for point-of-care testing of ischemic strokes. Lab Chip. 2024;24:4253–63. 10.1039/D4LC00398E. 39118539 10.1039/d4lc00398e

[107] Chen C, Ran B, Liu B, Liu X, Zhang Z, Li Y, et al. Multiplexed detection of biomarkers using a microfluidic chip integrated with mass-producible micropillar array electrodes. Anal Chim Acta. 2023;1272:341450. 10.1016/j.aca.2023.341450. 37355325 10.1016/j.aca.2023.341450

[108] Sierra T, Jang I, Noviana E, Crevillen AG, Escarpa A, Henry CS. Pump-free microfluidic device for the electrochemical detection of α1 -acid glycoprotein. ACS Sens. 2021;6:2998–3005. 10.1021/acssensors.1c00864. 34350757 10.1021/acssensors.1c00864

[109] Xu H, Hang Y, Wu Z, Lei X, Deng J, Yang J. Capillary-driven microchip integrated with nickel phosphide hybrid-modified electrode for the electrochemical detection of glucose. Anal Chim Acta. 2024;1316:342882. 10.1016/j.aca.2024.342882. 38969418 10.1016/j.aca.2024.342882

[110] Gutiérrez-Capitán M, Sanchís A, Carvalho EO, Baldi A, Vilaplana L, Cardoso VF, et al. Engineering a point-of-care paper-microfluidic electrochemical device applied to the multiplexed quantitative detection of biomarkers in sputum. ACS Sens. 2023;8:3032–42. 10.1021/acssensors.3c00523. 37467113 10.1021/acssensors.3c00523 PMC10463273

[111] Zhao P, Zuo J, Huang C, Hu Q, Zhang J, Xu J, et al. Biomimetic nanozyme-based electrochemical sensor integrated with microfluidic cell for on-site uric acid detection. Microchem J. 2024;207:112114. 10.1016/j.microc.2024.112114.

[112] Vinoth R, Sangavi P, Nakagawa T, Jayaraman M, Mohan AMV. All-in-one microfluidic device with an integrated porous filtration membrane for on-site detection of multiple salivary biomarkers. Sens Actuat B-Chem. 2023;379:133214. 10.1016/j.snb.2022.133214.

[113] Pungjunun K, Yakoh A, Chaiyo S, Praphairaksit N, Siangproh W, Kalcher K, et al. Laser engraved microapillary pump paper-based microfluidic device for colorimetric and electrochemical detection of salivary thiocyanate. Microchim Acta. 2021;188:140. 10.1007/s00604-021-04793-2. 10.1007/s00604-021-04793-2 33772376

[114] Yuksel M, Luo W, McCloy B, Mills J, Kayaharman M, Yeow JTW. A precise and rapid early pregnancy test: development of a novel and fully automated electrochemical point-of-care biosensor for human urine samples. Talanta. 2023;254:124156. 10.1016/j.talanta.2022.124156. 36525867 10.1016/j.talanta.2022.124156

[115] Fava EL, Martimiano Do Prado T, Almeida Silva T, Cruz De Moraes F, Censi Faria R, Fatibello‐Filho O. New disposable electrochemical paper‐based microfluidic device with multiplexed electrodes for biomarkers determination in urine sample. Electroanal. 2020;32:1075–1083. 10.1002/elan.201900641

[116] Fava EL, Silca TA, Prado TMD, Moraes FC, et al. Electrochemical paper-based microfluidic device for high throughput multiplexed analysis. Talanta. 2019;203:280–86. 10.1016/j.talanta.2019.05.081. 10.1016/j.talanta.2019.05.081 31202339

[117] Wang W, Ding S, Wang Z, Lv Q, Zhang Q. Electrochemical paper-based microfluidic device for on-line isolation of proteins and direct detection of lead in urine. Biosens Bioelectron. 2021;187:113310. 10.1016/j.bios.2021.113310. 34020224 10.1016/j.bios.2021.113310

